# The performance of sCD25 and CTLs degranulation test for screening patients with primary hemophagocytic lymphohistiocytosis: a large-scale multicenter study in China

**DOI:** 10.3389/fimmu.2025.1616541

**Published:** 2025-07-02

**Authors:** Wenshuai Zheng, Zhaoguang Chen, Yibo Cai, Zongze Wu, Zhenlan Du, Xiaohong Li, Beicheng Li, Shibin Zhu, Hongmei Ning

**Affiliations:** ^1^ Department of Hematology, Hainan Hospital of Chinese People’s Liberation Army (PLA) General Hospital, Sanya, Hainan, China; ^2^ Hematology and Oncology Department of Pediatrics, The Seventh Medical Center of Chinese People’s Liberation Army (PLA) General Hospital, Beijing, China; ^3^ Senior Department of Hematology, The Fifth Medical Center of Chinese People’s Liberation Army (PLA) General Hospital, Beijing, China; ^4^ Department of Hematology, The Fourth Medical Center of Chinese People’s Liberation Army (PLA) General Hospital, Beijing, China

**Keywords:** hemophagocytic lymphohistiocytosis, sCD25, cytotoxic T lymphocytes degranulation test, screening test, China

## Abstract

**Background:**

Hemophagocytic lymphohistiocytosis can be classified as primary HLH (pHLH) and secondary HLH. The early identify of pHLH can facilitate successful treatment. The rapid screening test greatly facilitated the diagnostic process of pHLH. However, there are rare studies to explore the ability of sCD25 and cytotoxic T lymphocytes (CTLs) degranulation test to rapidly screen pHLH.

**Methods:**

We validated the accuracies of sCD25 and CTLs degranulation test in screening pHLH in 1224 clinically confirmed or suspected HLH patients through calculating area under the curve (AUC) and diagnostic parameters at optimal threshold.

**Results:**

The sensitivities of sCD25 to identify pHLH with defects in cytotoxic pathway genes was 75.9%, with specificities of 57.3% and AUC of 0.640. The sensitivities of CTLs degranulation test to identify pHLH with defects in degranulation pathway genes was 87.0%, with specificities of 69.0% and AUC of 0.794. The sensitivities of combination of sCD25 and CTLs degranulation test to identify pHLH with defects in cytotoxic pathway genes was 90.0%, with specificities of 69.8% and AUC of 0.833.

**Conclusion:**

For identifying pHLH, CTLs degranulation test is more sensitive and no less specific than sCD25 and the combination of sCD25 and CTLs degranulation test can improve the accuracy further.

## Introduction

Hemophagocytic lymphohistiocytosis (HLH) is a rare, potentially fatal disorder characterized by a dysregulated activation of cytotoxic T lymphocytes, natural killer (NK) cells, and macrophages resulting in hyper-inflammation and immune-mediated injury of multiple organ systems (1). Historically, HLH is often classified as primary HLH (pHLH) and secondary HLH (sHLH) according to the etiology (2).

pHLH is a heritable disease conferred by genetic variations in several genes. Familial HLH (FHL) is the main form of pHLH, which was caused by mutations in *PRF1* or *UNC13D*, *STX11*, and *STXBP2* (3–6). In addition, primary immunodeficiency syndromes, caused by mutations in *LYST*, *RAB27A*, and *AP3B1*, are also associated with the development of HLH (7–9). Apart from *PRF1*, all of these genes are involved in the degranulation pathway of cytotoxic lymphocytes, which influences the precise processes of docking, priming, and fusion of the cytotoxic lymphocytes to the target cell. *PRF1* encodes perforin, which is a key cargo of cytolytic vesicles.

The early differentiation between pHLH and sHLH has important clinical implications. Because although aggressive immunosuppressive therapy is required for both pHLH and sHLH in initial period (10, 11), the post-remission management of pHLH and sHLH are variable. For pHLH, allogeneic hematopoietic stem cell transplantation (allo-HSCT) should be considered speedily (12–14), while the treatment should be focused on eliminating the secondary triggers for sHLH. Thus, rapid identification of pHLH can accelerate the initiation of allo-HSCT preparation.

Screening test that assesses cellular phenotype and function can greatly facilitate the diagnostic process, as they can quickly diagnosis and categorize different inherited forms of HLH, and guide sequencing efforts. For example, measuring intracytoplasmic perforin expression in cytotoxic lymphocytes has been shown to be a reliable way to screen patient with biallelic mutation in PRF1 (15, 16). The NK cell degranulation test has high sensitivity and specificity in screening pHLH with genetic defects in degranulation pathway (*UNC13D*, *STXBP2*, *STX11*, *LYST*, *RAB27A*, and *AP3B1*) (15, 17, 18). Nonetheless, a significant proportion of patients with sHLH presents with abnormal NK cells degranulation function, while certain pHLH exhibit normal NK cells degranulation function (17, 19). Another way to screen pHLH with genetic defects in degranulation pathway is to measure cytotoxic T lymphocytes (CTLs) degranulation function. However, there are rare studies to validated the performance of CTLs degranulation test in identifying pHLH (19, 20), In addition, there is no large study to evaluate its diagnostic performance in China, considering that the pattern of genetic mutations in pHLH varies significantly in different ethnic groups (21–23). Furthermore, it has not been evaluated that whether sCD25, also known as soluble interleukin-2 receptor alpha chain, as one of the eight HLH-2004 diagnostic criteria (24), has ability to screen pHLH.

In this report, we aimed to validate the accuracies of sCD25 and CTLs degranulation test in screening pHLH with biallelic variants in cytotoxic pathway genes (*PRF1*, *UNC13D*, *STXBP2*, *STX11*, *RAB27A*, *LYST*, and *AP3B1*).

## Patients and methods

### Patient and samples

Medical records of patients with clinically confirmed HLH [meeting five or more HLH-2004 diagnostic criteria (24)] or suspected HLH (meeting four or less HLH-2004 diagnostic criteria) were retrieved from the Fourth, Fifth and Seventh Medical Centers of Chinese PLA General Hospital (all at Beijing, China), and Hainan Hospital of Chinese PLA General Hospital (Sanya, China) for the period spanning January 2014 to September 2024. We reviewed the clinical information, immunological testing data, and targeted next-generation sequencing (t-NGS) results. Patients with biallelic variants or hemizygous mutations in pHLH-associated genes were defined as pHLH patients. The study protocol was reviewed and approved by the Ethical Committee of all participating four hospitals. All procedures involving human subjects were conducted in accordance with the international ethical standards and the Helsinki Declaration.

### Targeted generation sequencing

The t-NGS-based panel covers 7 pHLH-associated genes. Details of these genes are provided in **Supplementary Table 1**. Genomic DNA was extracted from peripheral blood or bone marrow samples using the QIAsymphony DNA Mini Kit (Qiagen, Hilden, Germany). Libraries were constructed using the QIAseq FX DNA Library Kit (Qiagen, Hilden, Germany). DNA sequencing was performed on an Illumina NovaSeq6000 system (Illumina, San Diego, CA, USA) with DNA input of 100 ng. Sequence reads were aligned to the human reference ge-nome (NCBI build 37/hg19).

### CTLs degranulation testing and sCD25

For analysis of CTLs degranulation, PBMCs were isolated from peripheral blood and were stimulated with K562 target cells in a specific effector-to-target ratio. Unstimulated PBMCs served as control. Flow cytometry (Dxflex; Beckman Coulter, Brea, CA, USA) was used to analyze CTLs degranulation with anti-CD3-FITC, anti-CD8-APC, anti-CD56-PC5.5, and anti-CD107a-PE (all from Invigentech, Brea, CA, USA). CD3^+^CD8^+^ T cells were gated and surface CD107a expression was quantified. CD107a Δ mean fluorescence intensity (ΔMFI) was calculated as the ratio of CD107a MFI in K562-stimulated samples to unstimulated controls. Results were classified as normal (CD107a ΔMFI ≥ 2.8) or defective (CD107a ΔMFI < 2.8).

For analysis of sCD25, serum was isolated from peripheral blood. The serum sCD25 concentration was determined by enzyme-linked immunosorbent assay (MultiskanFC; Thermo Fisher Scientific, Waltham, MA, USA) according to the standard curve established by the standard substance (normal range of sCD25 < 6400 pg/ml).

If a patient had multiple results for a given immunologic assay, only the first valid result was included in the analysis.

### Bioinformatics and statistical analysis

The Genome Aggregation Database (gnomAD; http://gnomad.broadinstitute.org/) was used to obtain the frequencies of variants in the general Eastern Asian population. Sequence variants were analyzed using the Sorting Intolerant From Tolerant (SIFT), Polymorphism Phenotype v2 (Polyphen-2) and Protein Variation Effect Analyzer (PROVEAN) tools (25–27). Variants were categorized according to the American College of Medical Genetics and Genomics (ACMG) guidelines (28). Only variants classified as pathogenic, likely pathogenic or uncertain significance were included in the analysis. To further narrow down the number of candidate variants, we restricted our analysis to variants with minor allele frequency (MAF) of less than 5% in the general Eastern Asian population and novel variants in the Genome Aggregation Database. This approach has previously been successfully employed to identify candidate gene variants in other autosomal recessive diseases (29).

Non-normally distributed data was present as median and interquartile ranges (IQR). The two sample comparison of non-normally distributed data were performed using Mann-Whitney U test. We evaluated the sensitivity, specificity, positive predictive value (PPV), and negative predictive value (NPV) of CTLs degranulation test and sCD25 for discriminating pHLH patients, based on the laboratory-generated normal ranges for each test. We also performed receiver operating characteristic (ROC) curves to determine the optimal threshold of each test that would identify pHLH patients with maximum sensitivity and specificity.

We created a model to assess the performance of combination of CTLs degranulation test and sCD25 for screening pHLH patients. To produce areas under ROC curves, we fit a logistic regression model with a linear and quadratic term for each marker under consideration. The coefficients obtained from the regression model were applied to the values of the marker for each patient to obtain the fitted values. Sensitivity and specificity for the fitted values were calculated and plotted in the ROC curves. All statistical analyses were performed by R Statistical Software (R Foundation for Statistical Computing) using the R package pROC (30). All P values were calculated as two-sided and P values less than 0.05 were considered statistically significant (indicated as *P < 0.05, **P < 0.01, ***P < 0.001, and ****P < 0.0001).

## Results

### General information

A total of 1,224 patients with pHLH-associated t-NGS results were included. Among these patients, 675 (55.2%) were male and 549 (44.8%) were female. Among 1,166 patients with known age of onset, the median age of onset was 24 years (IQR, 5–45 years). Of 1,224 patients, 78 patients had biallelic variants in cytotoxic pathway genes, including 30 patients with variants in UNC13D, 30 patients with variants in PRF1, 14 patients with variants in LYST, 2 patients with variants in STXBP2, and 2 patients with variants in RAB27A, 236 patients had single/digenic/polygenic heterozygous variants in cytotoxic pathway genes, and 910 patients without any variants associated cytotoxic pathway genes. For the test results, 832 patients had the results of sCD25, 458 patients had the results of CTLs degranulation test, and 225 patients had the results of sCD25 and CTLs degranulation test simultaneously. Detailed demographic and clinical information of all patients are provided in **Supplementary Table S2–4**.

### The accuracy of sCD25 in discriminating pHLH was limited

**Figure 1:**
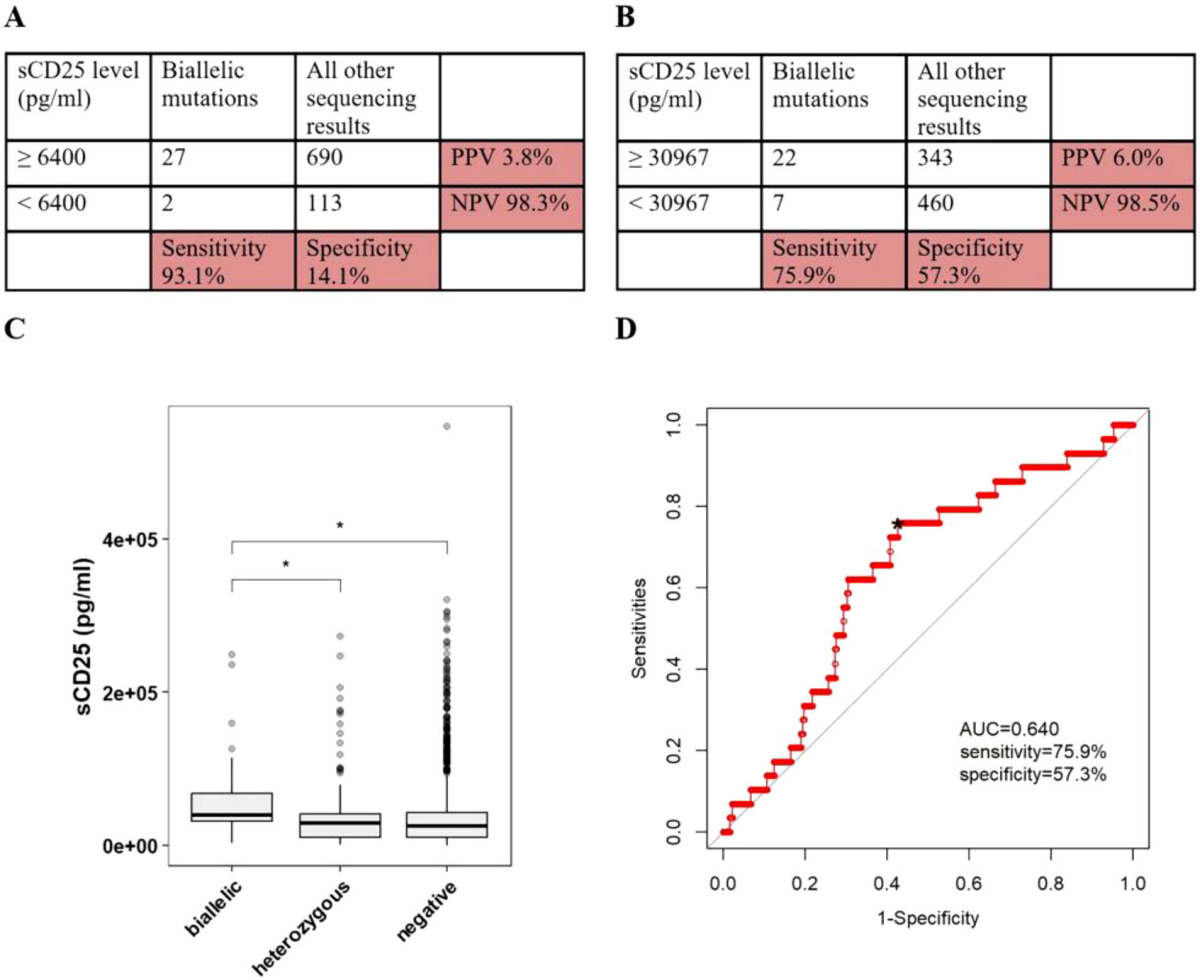
The accuracy of sCD25 in discriminating pHLH with biallelic variants in PRF1, UNC13D, STX11, STXBP2, RAB27A, LYST or AP3B1. **(A)** Sensitivity/specificity/PPV/NPV of high sCD25 in distinguishing patients with any biallelic variants from patients with all other sequencing results. **(B)** Sensitivity/specificity/PPV/NPV of the cutoff value of sCD25 in distinguishing patients with any biallelic variants from patients with all other sequencing results. **(C)** The conparision of sCD25 among patients with differen sequencing results. **(D)** ROC curve of sCD25. *optimal cutoff value.

When patients with the results of sCD25 were grouped according to variants forms of cytotoxic pathway genes, 29 patients had biallelic variants in cytotoxic pathway genes with a median sCD25 of 40028 pg/ml (IQR, 30987-67265), 105 patients had single/digenic/polygenic heterozygous variants in cytotoxic pathway genes with a median sCD25 of 29096 pg/ml (IQR, 10743-40774), and 698 patients had not any variants in cytotoxic pathway genes with a median sCD25 of 25482 pg/ml (IQR, 10474-42923). Patients with variants in PRF1 were classified as patients biallelic variants or patients with single/digenic/polygenic heterozygous variants according to the type of zygote. When performing a comparison between patients with biallelic variants and other groups, patients with biallelic variants had a higher sCD25 than that of patients with single/digenic/polygenic heterozygous variants (*P* = 0.020), and patients without any variants (*P* = 0.011) (**Figure 1C**).

Based on the laboratory-defined thresholds (6400 pg/ml), 27 of 29 patients (93.1%) with biallelic variants had higher sCD25, while 690 of 803 patients (85.9%) without biallelic variants had higher sCD25, in which 90 of 105 patients (85.7%) with single/digenic/polygenic heterozygous variants had higher sCD25, 600 of 698 patients (86.0%) without any variants had higher sCD25. When using 6400 pg/ml as the cutoff values, the sensitivity of sCD25 in discriminating patients with biallelic variants in cytotoxic pathway genes from patients with all other sequencing results was 93.1%, specificity was 14.1%, PPV was 3.8%, and NPV was 98.3% (**Figure 1A**). ROC analysis was constructed to evaluate the diagnostic efficacy of sCD25. The optimal diagnostic threshold was 30976 pg/ml and the area under the curve (AUC) was 0.640, with a corresponding sensitivity, specificity, PPV and NPV of 75.9%, 57.3%, 6.0%, and 98.5%, respectively (**Figures 1B, D**).

### The accuracy of CTLs degranulation test in discriminating pHLH was good

**Figure 2:**
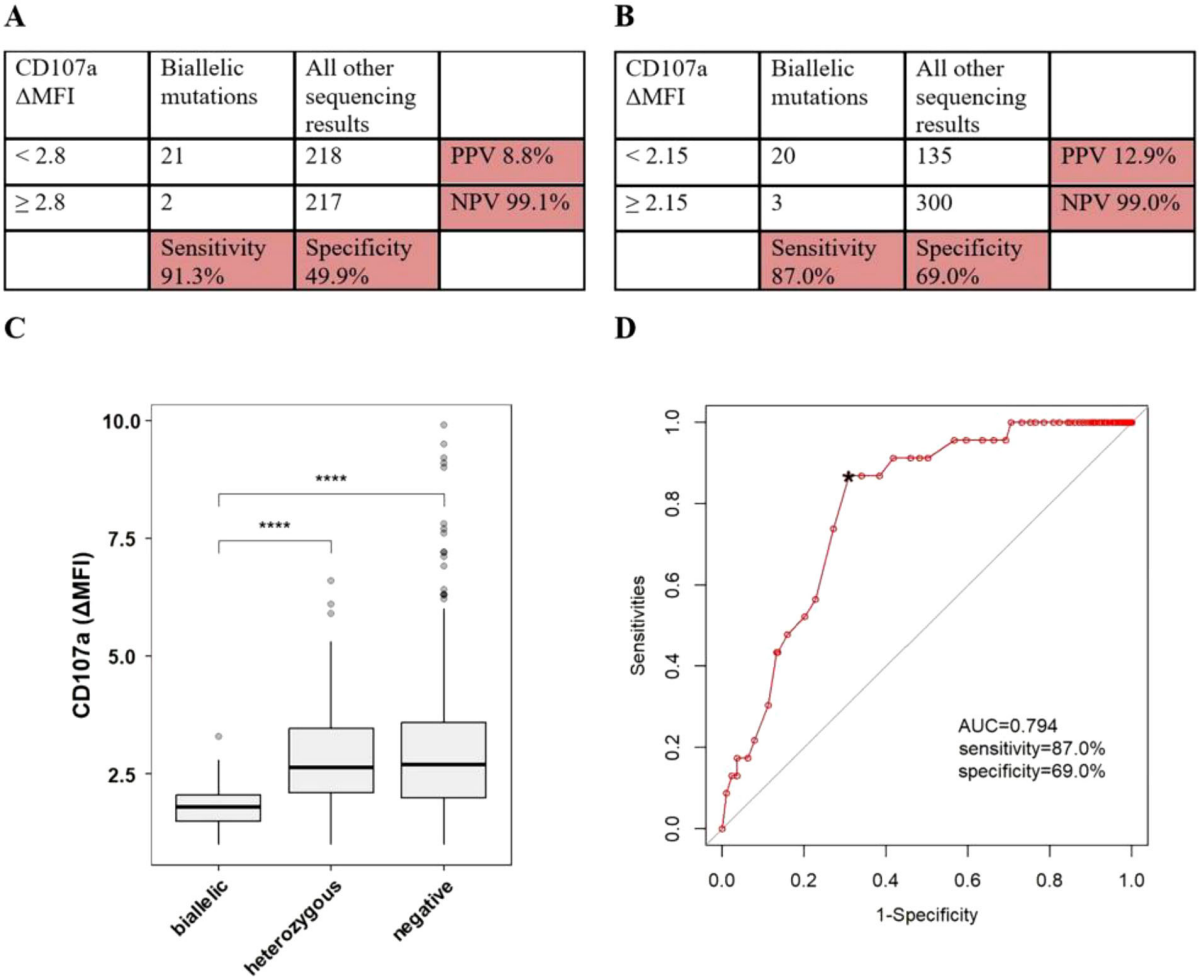
The accuracy of CTLs degranulation test in discriminating pHLH with biallelic variants in UNC13D, STX11, STXBP2, RAB27A, LYST or AP3B1. **(A)** Sensitivity/specificity/PPV/NPV of low CD107a ΔMFI in distinguishing patients with any biallelic variants from patients with all other sequencing results. **(B)** Sensitivity/specificity/PPV/NPV of the cutoff value of CD107a ΔMFI in distinguishing patients with any biallelic variants from patients with all other sequencing results. **(C)** The conparision of CD107a ΔMFI among patients with differen sequencing results. **(D)** ROC curve of CTLs degranulation test. *optimal cutoff value. ****P<0.0001.

When patients with the results of CTLs degranulation test were grouped according to carrying forms of variants in degranulation genes, 23 patients had biallelic variants in degranulation genes with a median CD107a ΔMFI of 1.8 (IQR, 1.5-2.05), 54 patients had single/digenic/polygenic heterozygous variants in degranulation pathway genes with a median CD107a ΔMFI of 2.65 (IQR, 2.1-3.475), and 381 patients had not any variants in degranulation genes with a median CD107a ΔMFI of 2.7 (IQR, 2-3.6). Considering the limited influence of PRF1 on degranulation pathway of cytotoxic lymphocytes, patients with variants in PRF1 were classified as patients without any variants in degranulation genes. When performing a comparison between patients with biallelic variants and other groups, patients with biallelic variants had a higher sCD25 relative to patients with single/digenic/polygenic heterozygous variants (*P* < 0.001) and patients without any variants (*P* < 0.001) (**Figure 2C**).

Based on the laboratory-defined thresholds (2.8), 21 of 23 patients (91.3%) with biallelic variants had lower CD107a ΔMFI, while 218 of 435 patients (50.1%) without biallelic variants had lower CD107a ΔMFI, in which 27 of 54 patients (50.0%) with single/digenic/polygenic heterozygous variants had lower CD107a ΔMFI, 191 of 381 (50.1%) without any variants had lower CD107a ΔMFI. When using < 2.8 as the cutoff values, the sensitivity of CTLs degranulation test for discriminating patients with biallelic variants in degranulation genes from patients with all other sequencing results was 91.3%, specificity was 49.9%, PPV was 8.8%, and NPV was 99.1% (**Figure 2A**). ROC analysis was constructed to evaluate the diagnostic efficacy of CTLs degranulation test. The optimal diagnostic threshold was 2.15 and the area under the curve (AUC) was 0.794, with a corresponding sensitivity, specificity, PPV and NPV of 87.0%, 69.0%, 12.9%, and 99.0%, respectively (**Figures 2B, D**).

### The combination of sCD25 and CTLs degranulation test improved the accuracy in discriminating pHLH

**Figure 3:**
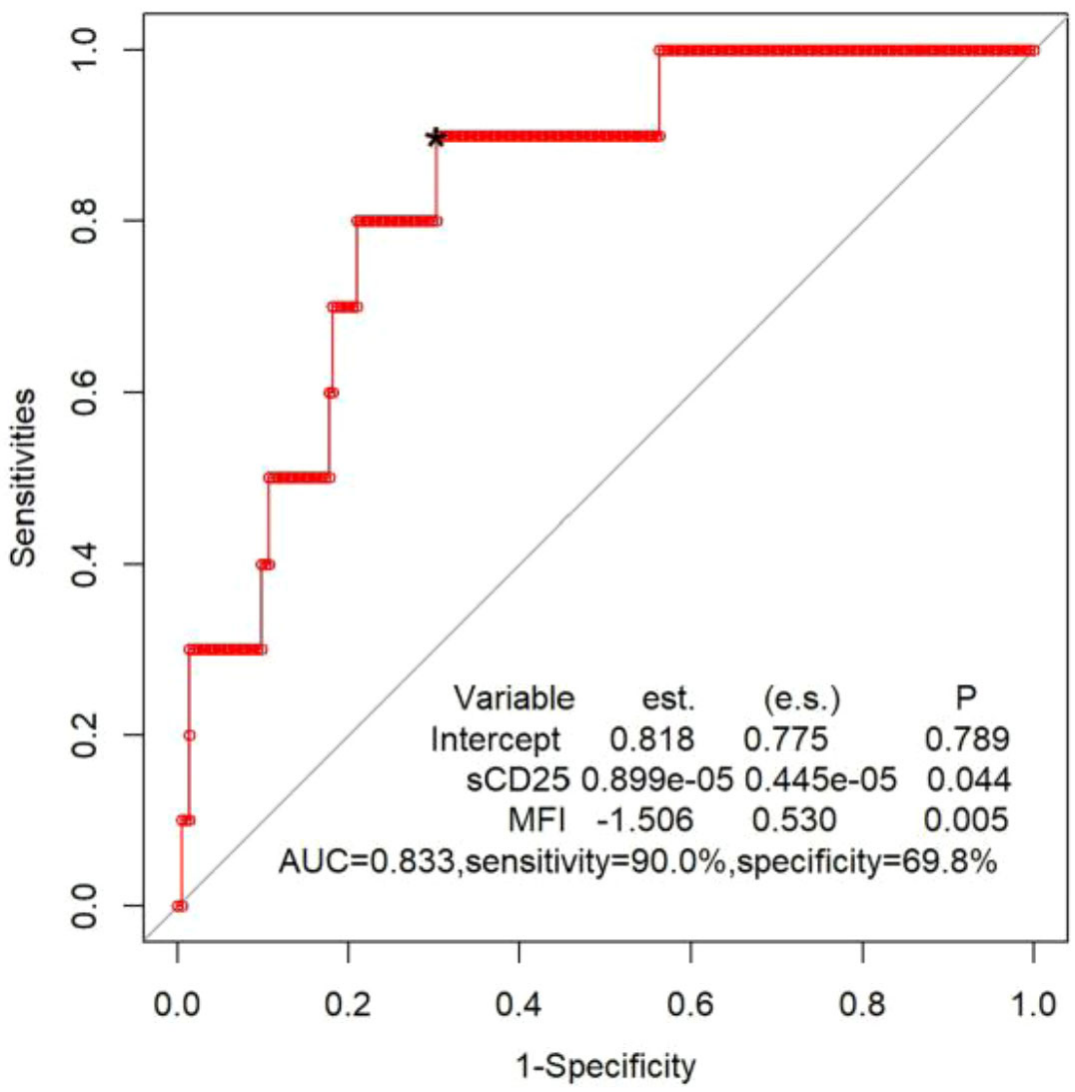
ROC curve of logistic regression model, combining sCD25 and and CTLs degranulation test to distinguish patients with any biallelic variants in UNC13D, STX11, STXBP2, RAB27A, LYST or AP3B1 from patients with all other sequencing results. est., coefficient of the logistic regression model; *optimal cutoff value for the fitted values obtained from the logistic regression model; s.e., standard error for the coefficient.

Lastly, we analysed 225 patients who had the results of sCD25 and CTLs degranulation test simultaneously. Ten of 225 patients had biallelic variants in cytotoxic pathway genes. By applying patient values to a logistic regression model and generating an ROC figure, the combination of sCD25 and CTLs degranulation test yielded a sensitivity of 90.0% and specificity of 69.8%, with an AUC of 0.833 (**Figure 3**).

## Discussion

In the HLH-2004 diagnostic criteria, Histiocyte Society pointed out that the demonstration of pathogenic mutations of HLH-associated genes is the gold standard for pHLH diagnosis. However, a molecular diagnosis of pHLH is often time-consuming and costly. The rapid immunological tests, before the results of genetic sequence, can make a rapid prediction for pHLH and accelerate preparations for eventual allo-HSCT. In the present study, we reported the accuracies of sCD25 and CTLs degranulation test in discriminating patients with biallelic variants in cytotoxic pathway genes. To our knowledge, this is the first study to assess the performance of sCD25 in screening pHLH. This study is also the largest to assess the performance of CTLs degranulation test in discriminating pHLH.

sCD25, as a marker of T-cell activity (31), expressed high sensitivity in diagnosing HLH and comprises one of the eight diagnostic criteria of HLH-2004 trial (32, 33). In adults, Yoon et al. reported that the performance of sCD25 in discriminating HLH was high, with a sensitivity of more than 90% and a specificity of 80%. Hayden et al. showed that the AUC of sCD25 in diagnosing HLH reached 0.90 (32, 33). In addition to the diagnostic value for HLH, sCD25 also showed the ability to distinguish different types of HLH. Zhao W, et al. reported that sCD25 in tumor-related HLH was significantly higher than that in non-tumor-related HLH. When using > 23000ng/ml as the cutoff value, the sensitivity and specificity of sCD25 in discriminating tumor-related HLH from non-tumor-related HLH were 70.7% and 66.8%, respectively (34). However, the performance of sCD25 in discriminating pHLH from sHLH has not been evaluated. In our study, we found that sCD25 of patients with biallelic variants in cytotoxic pathway genes was significantly higher than that of other patients. So, we used the laboratory-defined thresholds (6400 pg/ml) as the cutoff value to discriminate patients with biallelic variants in cytotoxic pathway genes from patients with all other sequencing results, the sensitivity and specificity of sCD25 were 93.1% and 14.1%, respectively. When using optimal diagnostic threshold (30976 pg/ml) determined by ROC curve as the cutoff value, the sensitivity and specificity of sCD25 were 75.9% and 57.5%, respectively, with an AUC of 0.643. This result indicated that the performance of sCD25 in discriminating pHLH from sHLH was not well. Nevertheless, the sCD25 does possess an excellent negative predictive value (> 98%), and thus is helpful for ruling out pHLH.

CD107a, also known as lysosome-associated membrane protein-1, is the major component of vesicle membrane proteins in CTLs and NK cells. During the cytotoxic activity of CTLs and NK cells, cytolytic granules are released and CD107a are transported to the cell surface. Therefore, the level of CD107a expression is well correlated with degranulation activity of CTLs and NK cells. The release of cytolytic granules involves multiple degranulation pathway genes that regulate vesicle transport. So, the evaluation of CTLs or NK cells degranulation function using flow cytometric measurement of CD107a upregulation can quickly identify pHLH with degranulation pathway genes deficiency. NK cells degranulation test was the most common method to detect the degranulation function and had showed high accuracy in identifying pHLH with genetic defects in degranulation pathway (15, 17, 18). However, studies associated with CTLs degranulation test are rare and the scale is limited (19, 20). In our study, we validated the performance of CTLs degranulation test in identifying pHLH with genetic defects in degranulation pathway. When using optimal diagnostic threshold (2.15) determined by ROC curve as the cutoff value, the sensitivity and specificity of CTLs degranulation test were 87.0% and 69.0%, respectively, with an AUC of 0.794. The accuracy of CTLs degranulation test in discriminating pHLH was slight lower than that of NK cell degranulation test reported by Rubin et al. (sensitivity, 93.8%; specificity, 73.0%; AUC, 0.860). In addition, we explored whether the combination of sCD25 and CTLs degranulation test can improve the accuracy in discriminating pHLH. The results showed that compared with CTLs degranulation test, the combination of sCD25 and CTLs degranulation test improved the sensitivity from 87.0% to 90.0% while the specificity remain stable, with an AUC of 0.833.

The strength of our study is that the design background conforms to clinical practice, in which cases include both clinically suspected and confirmed HLH. Previous studies, exploring the ability of different methods to distinguish pHLH from sHLH, main included patients with clinical confirmed HLH. However, in clinical practice, these tests were not only applicated to patients who met the HLH-2004 diagnostic criteria but also to patients with clinical suspected HLH. In addition, the large scale of the cohort is also key strengths of this study. Of course, our study also has limitation that we limited our analysis to only immunologic test and sequencing results due to the absent of other clinical information.

In summary, CTLs degranulation test had a high accuracy in screening pHLH with genetic defects in degranulation pathway. Compared with CTLs degranulation test, the diagnostic value of sCD25 was less sensitive and no more specific, but it possessed an excellent negative predictive value to rule out pHLH. Based on our data, CTLs degranulation test was a reliable method to screen pHLH with genetic defects in degranulation pathway and the combination of sCD25 and CTLs degranulation test can improve the accuracy.

## Data Availability

The original contributions presented in the study are included in the article/**Supplementary Material**. Further inquiries can be directed to the corresponding authors.
