# Supplementary material for: The performance of sCD25 and CTLs degranulation test for screening patients with primary hemophagocytic lymphohistiocytosis: a large-scale multicenter study in China
Source: Front Immunol. 2025 Jul 2;16:1616541. doi: 10.3389/fimmu.2025.1616541 (PMC12263684; doi:10.3389/fimmu.2025.1616541)
Supplement: Supplementary file 1 [file DataSheet1.docx]

Table S1. Seven genes that are tested in targeted generation sequencing panel.

| **Symbol**  **(Transcript)** | **Gene** **OMIM** | **Associated** **OMIM** **Disease** | **Inheritance** |
| --- | --- | --- | --- |
| AP3B1  (NM_003664) | 603401 | Hermansky-Pudlak syndrome 2 | AR |
| LYST  (NM_000081) | 606897 | Chediak-Higashi syndrome | AR |
| PRF1  (NM_001083116) | 170280 | Hemophagocytic lymphohistiocytosis, familial, 2; Aplastic anemia; Lymphoma, non-Hodgkin | AR |
| RAB27A  (NM_004580) | 603868 | Griscelli syndrome, type 2 | AR |
| STX11  (NM_003764) | 605014 | Hemophagocytic lymphohistiocytosis, familial, 4 | AR |
| STXBP2  (NM_006949) | 601717 | Hemophagocytic lymphohistiocytosis, familial, 5 | AR |
| UNC13D  (NM_199242) | 608897 | Hemophagocytic lymphohistiocytosis, familial, 3 | AR |

AR autosomal recessive.

Table S2. Patients with primary hemophagocytic lymphohistiocytosis.

| Patients | Age at testing, year | Sex | Gene | Zygote | sCD25  pg/ml | CTLs degranulation (CD107a ΔMFI) | Frequency in gnomAD*, % | Polyphen2 | SIFT | PROVEAN | ACMG  classification |
| --- | --- | --- | --- | --- | --- | --- | --- | --- | --- | --- | --- |
| 1 | 41 | Male | UNC13D | Hom | NA | NA | 0.371 | probably damaging | Damaging | Deleterious | Possibly pathogenic |
| 2 | 1.7 | Female | UNC13D | Hom | NA | 2 | 0.371 | probably damaging | Damaging | Deleterious | Possibly pathogenic |
| 3 | 63 | Male | UNC13D | Hom | NA | NA | 0.371 | probably damaging | Damaging | Deleterious | Possibly pathogenic |
| 4 | 30 | Female | UNC13D | Hom | NA | 2.1 | 0.371 | probably damaging | Damaging | Deleterious | Possibly pathogenic |
| 5 | 18 | Male | UNC13D | Hom | 32257 | NA | 0.371 | probably damaging | Damaging | Deleterious | Possibly pathogenic |
| 6 | 2 | Male | UNC13D | Hom | NA | 1.4 | 0.371 | probably damaging | Damaging | Deleterious | Possibly pathogenic |
| 7 | 0.2 | Male | UNC13D | Hom | NA | NA | NA | NA | NA | NA | Possibly pathogenic |
| 8 | 2 | Male | UNC13D | Hom | 159730 | 1.1 | NA | probably damaging | Damaging | Deleterious | Possibly pathogenic |
| 9 | 6 | Female | UNC13D | Hom | NA | 2.4 | 0.0057 | probably damaging | Damaging | Deleterious | Possibly pathogenic |
| 10 | 1 | Female | UNC13D | Het | NA | NA | NA | NA | NA | NA | Pathogenic |
|  |  |  | UNC13D | Het |  |  | NA | NA | NA | NA | Possibly pathogenic |
| 11 | 11 | Male | UNC13D | Het | 40867 | NA | 0.005441 | probably damaging | Damaging | Deleterious | Uncertain significance |
|  |  |  | UNC13D | Het |  |  | NA | NA | NA | NA | Possibly pathogenic |
| 12 | 1 | Male | UNC13D | Het | NA | NA | 0.005441 | probably damaging | Damaging | Deleterious | Uncertain significance |
|  |  |  | UNC13D | Het |  |  | 0 | benign | Damaging | Deleterious | Uncertain significance |
| 13 | 2.3 | Male | UNC13D | Het | NA | 1.24 | 0.1607 | NA | NA | NA | Uncertain significance |
|  |  |  | UNC13D | Het |  |  | 0.1025 | benign | Tolerated | Neutral | Uncertain significance |
| 14 | 0.1 | Male | UNC13D | Het | 39900 | NA | NA | NA | NA | NA | Pathogenic |
|  |  |  | UNC13D | Het |  |  | 0.00544 | NA | NA | NA | Possibly pathogenic |
| 15 | 4 | Male | UNC13D | Het | 235135 | 2.1 | 0 | NA | NA | NA | Pathogenic |
|  |  |  | UNC13D | Het |  |  | 0.0057 | probably damaging | Damaging | Deleterious | Possibly pathogenic |
| 16 | 25 | Female | UNC13D | Het | 114530 | 3.3 | 0 | NA | NA | NA | Pathogenic |
|  |  |  | UNC13D | Het |  |  | 0.02006 | possibly damaging | Damaging | Deleterious | Possibly pathogenic |
| 17 | 14 | Female | UNC13D | Het | NA | 1.8 | 0 | probably damaging | Damaging | Deleterious | Uncertain significance |
|  |  |  | UNC13D | Het |  |  | 0 | probably damaging | Damaging | Deleterious | Possibly pathogenic |
| 18 | 3 | Male | UNC13D | Het | NA | NA | 0.01632 | benign | Tolerated | Neutral | Uncertain significance |
|  |  |  | UNC13D | Het |  |  | NA | benign | Tolerated | Neutral | Uncertain significance |
| 19 | 4 | Male | UNC13D | Het | NA | 1 | 1.609 | benign | Tolerated | Neutral | Uncertain significance |
|  |  |  | UNC13D | Het |  |  | 0.371 | probably damaging | Damaging | Deleterious | Possibly pathogenic |
| 20 | 11 | Female | UNC13D | Het | NA | 1 | 1.609 | benign | Tolerated | Neutral | Uncertain significance |
|  |  |  | UNC13D | Het |  |  | 0.05766 | probably damaging | Damaging | Neutral | Uncertain significance |
| 21 | 16 | Male | UNC13D | Het | NA | 1.7 | 0.00544 | NA | NA | NA | Possibly pathogenic |
|  |  |  | UNC13D | Het |  |  | 0.371 | probably damaging | Damaging | Deleterious | Possibly pathogenic |
| 22 | 31 | Male | UNC13D | Het | NA | NA | NA | NA | NA | NA | Pathogenic |
|  |  |  | UNC13D | Het |  |  | 0.02006 | possibly damaging | Damaging | Deleterious | Possibly pathogenic |
| 23 | 3 | Male | UNC13D | Het | 248995 | NA | NA | probably damaging | Damaging | Deleterious | Uncertain significance |
|  |  |  | UNC13D | Het |  |  | NA | NA | NA | NA | Pathogenic |
| 24 | 31 | Male | UNC13D | Het | NA | NA | NA | NA | NA | NA | Possibly pathogenic |
|  |  |  | UNC13D | Het |  |  | 0.371 | probably damaging | Damaging | Deleterious | Possibly pathogenic |
| 25 | 3 | Female | UNC13D | Het | NA | 2 | 0.04812 | benign | Tolerated | Neutral | Uncertain significance |
|  |  |  | UNC13D | Het |  |  | NA | NA | NA | NA | Uncertain significance |
| 26 | 9.5 | Female | UNC13D | Het | NA | NA | 0.04812 | benign | Tolerated | Neutral | Uncertain significance |
|  |  |  | UNC13D | Het |  |  | 0.00628 | probably damaging | Damaging | Deleterious | Uncertain significance |
| 27 | 39 | Male | UNC13D | Het | 40028 | NA | 0.371 | probably damaging | Damaging | Deleterious | Possibly pathogenic |
|  |  |  | UNC13D | Het |  |  | 0.007113 | NA | NA | NA | Pathogenic |
| 28 | 0.2 | Female | UNC13D | Het | 24046 | NA | NA | NA | NA | NA | Possibly pathogenic |
|  |  |  | UNC13D | Het |  |  | 0.007113 | NA | NA | NA | Pathogenic |
| 29 | 2.3 | Female | UNC13D | Het | NA | 2 | 0.0052 | NA | NA | NA | Uncertain significance |
|  |  |  | UNC13D | Het |  |  | NA | NA | NA | NA | Uncertain significance |
| 30 | 10.5 | Female | UNC13D | Het | NA | NA | 0 | probably damaging | Damaging | Deleterious | Uncertain significance |
|  |  |  | UNC13D | Het |  |  | 0.1025 | benign | Tolerated | Neutral | Uncertain significance |
| 31 | 12 | Female | PRF1 | Hom | 14721 | NA | 0.005836 | possibly damaging | Tolerated | Neutral | Uncertain significance |
| 32 | 10.6 | Male | PRF1 | Hom | 39455 | NA | 0 | possibly damaging | Damaging | Deleterious | Possibly pathogenic |
| 33 | 11 | Female | PRF1 | Hom | 64205 | 1.1 | 0.005439 | possibly damaging | Damaging | Deleterious | Pathogenic |
| 34 | 34 | Female | PRF1 | Hom | 88785 | 1.8 | 0.00503 | benign | Damaging | Deleterious | Pathogenic |
| 35 | 9 | Male | PRF1 | Hom | NA | NA | 0.00503 | benign | Damaging | Deleterious | Pathogenic |
| 36 | 10 | Male | PRF1 | Hom | NA | NA | 0.005437 | possibly damaging | Damaging | Deleterious | Pathogenic |
| 37 | 17 | Female | PRF1 | Hom | NA | NA | 0.005437 | possibly damaging | Damaging | Deleterious | Pathogenic |
| 38 | 7 | Male | PRF1 | Hom | 41261 | NA | 0.005437 | possibly damaging | Damaging | Deleterious | Pathogenic |
| 39 | 17 | Male | PRF1 | Hom | 35104 | NA | 0.005437 | possibly damaging | Damaging | Deleterious | Pathogenic |
| 40 | 9.2 | Female | PRF1 | Hom | 41135 | NA | 0.005437 | possibly damaging | Damaging | Deleterious | Pathogenic |
| 41 | 17 | Male | PRF1 | Hom | NA | NA | 0.005437 | possibly damaging | Damaging | Deleterious | Pathogenic |
| 42 | 10.9 | Female | PRF1 | Hom | 125520 | NA | 0.005437 | possibly damaging | Damaging | Deleterious | Pathogenic |
| 43 | 9 | Male | PRF1 | Hom | NA | NA | 0.005437 | possibly damaging | Damaging | Deleterious | Pathogenic |
| 44 | 55 | Male | PRF1 | Het | NA | NA | 0.005836 | possibly damaging | Tolerated | Neutral | Uncertain significance |
|  |  |  | PRF1 | Het |  |  | 0.05012 | benign | Tolerated | Neutral | Possibly pathogenic |
| 45 | 0.1 | Male | PRF1 | Het | NA | NA | 0.01675 | NA | NA | NA | Pathogenic |
|  |  |  | PRF1 | Het |  |  | 0.05012 | benign | Tolerated | Neutral | Possibly pathogenic |
| 46 | 28 | Male | PRF1 | Het | NA | NA | 0.01675 | NA | NA | NA | Pathogenic |
|  |  |  | PRF1 | Het |  |  | 0.05012 | benign | Tolerated | Neutral | Possibly pathogenic |
| 47 | 58 | Male | PRF1 | Het | NA | NA | 0.01675 | NA | NA | NA | Pathogenic |
|  |  |  | PRF1 | Het |  |  | 0.05012 | benign | Tolerated | Neutral | Possibly pathogenic |
| 48 | 59 | Male | PRF1 | Het | NA | NA | 0.01675 | NA | NA | NA | Pathogenic |
|  |  |  | PRF1 | Het |  |  | 0.05012 | benign | Tolerated | Neutral | Possibly pathogenic |
| 49 | 0.1 | Female | PRF1 | Het | NA | NA | 0.01675 | NA | NA | NA | Pathogenic |
|  |  |  | PRF1 | Het |  |  | 0.05012 | benign | Tolerated | Neutral | Possibly pathogenic |
| 50 | 0.1 | Male | PRF1 | Het | NA | NA | 0.01675 | NA | NA | NA | Pathogenic |
|  |  |  | PRF1 | Het |  |  | NA | NA | NA | NA | Pathogenic |
| 51 | 34 | Male | PRF1 | Het | 30987 | NA | 0.01675 | NA | NA | NA | Pathogenic |
|  |  |  | PRF1 | Het |  |  | 0.04896 | probably damaging | Damaging | Deleterious | Uncertain significance |
| 52 | 3 | Male | PRF1 | Het | 3758 | NA | NA | benign | Tolerated | Neutral | Uncertain significance |
|  |  |  | PRF1 | Het |  |  | 0 | possibly damaging | Damaging | Deleterious | Uncertain significance |
| 53 | 27 | Male | PRF1 | Het | NA | NA | 0.0641 | probably damaging | Damaging | Deleterious | Uncertain significance |
|  |  |  | PRF1 | Het |  |  | 0.05012 | benign | Tolerated | Neutral | Possibly pathogenic |
| 54 | 11 | Male | PRF1 | Het | NA | NA | 0 | probably damaging | Damaging | Deleterious | Uncertain significance |
|  |  |  | PRF1 | Het |  |  | 0.005437 | possibly damaging | Damaging | Deleterious | Pathogenic |
| 55 | 26 | Male | PRF1 | Het | NA | NA | 0.04896 | probably damaging | Damaging | Deleterious | Uncertain significance |
|  |  |  | PRF1 | Het |  |  | 0.005471 | NA | NA | NA | Possibly pathogenic |
| 56 | 1 | Male | PRF1 | Het | 7139 | NA | 0 | benign | Tolerated | Neutral | Uncertain significance |
|  |  |  | PRF1 | Het |  |  | 0.1921 | benign | Damaging | Neutral | Uncertain significance |
| 57 | 12 | Female | PRF1 | Het | NA | NA | 0 | possibly damaging | Damaging | Deleterious | Possibly pathogenic |
|  |  |  | PRF1 | Het |  |  | 0.005439 | possibly damaging | Damaging | Deleterious | Pathogenic |
| 58 | 40 | Male | PRF1 | Het | NA | NA | 0 | probably damaging | Damaging | Deleterious | Pathogenic |
|  |  |  | PRF1 | Het |  |  | 0.1921 | benign | Damaging | Neutral | Uncertain significance |
| 59 | 8 | Female | PRF1 | Het | 50202 | NA | NA | probably damaging | Damaging | Deleterious | Uncertain significance |
|  |  |  | PRF1 | Het |  |  | 0 | NA | NA | NA | Possibly pathogenic |
| 60 | 1.9 | Female | PRF1 | Het | 67265 | NA | NA | possibly damaging | Damaging | Deleterious | Uncertain significance |
|  |  |  | PRF1 | Het |  |  | NA | probably damaging | Damaging | Deleterious | Uncertain significance |
| 61 | 2 | Male | LYST | Het | 42366 | 1.5 | 0.005451 | possibly damaging | Damaging | Deleterious | Uncertain significance |
|  |  |  | LYST | Het |  |  | 0.06539 | benign | Tolerated | Neutral | Uncertain significance |
| 62 | 29 | Male | LYST | Het | NA | NA | 0.01634 | benign | Damaging | Neutral | Uncertain significance |
|  |  |  | LYST | Het |  |  | 0.06019 | benign | Damaging | Neutral | Uncertain significance |
| 63 | 7 | Female | LYST | Het | 2614 | 1.6 | 1.147 | benign | Tolerated | Neutral | Uncertain significance |
|  |  |  | LYST | Het |  |  | 0.08712 | benign | Tolerated | Neutral | Uncertain significance |
| 64 | 29 | Female | LYST | Het | 17301 | NA | 0 | probably damaging | Damaging | Deleterious | Uncertain significance |
|  |  |  | LYST | Het |  |  | 0.01088 | benign | Tolerated | Deleterious | Uncertain significance |
| 65 | 22 | Male | LYST | Het | 11292 | 1.6 | 0.1757 | benign | Damaging | Deleterious | Uncertain significance |
|  |  |  | LYST | Het |  |  | 0.01635 | benign | Tolerated | Neutral | Uncertain significance |
| 66 | 1.5 | Female | LYST | Het | NA | 2.8 | 0.01094 | benign | Tolerated | Deleterious | Uncertain significance |
|  |  |  | LYST | Het |  |  | 0 | possibly damaging | Tolerated | Neutral | Uncertain significance |
| 67 | 74 | Male | LYST | Het | 32710 | 1.9 | 0.03009 | benign | Tolerated | Deleterious | Uncertain significance |
|  |  |  | LYST | Het |  |  | 0.03012 | benign | Tolerated | Neutral | Uncertain significance |
| 68 | 33 | Female | LYST | Het | NA | NA | 0.03009 | benign | Tolerated | Deleterious | Uncertain significance |
|  |  |  | LYST | Het |  |  | 0.03012 | benign | Tolerated | Neutral | Uncertain significance |
| 69 | 28 | Female | LYST | Het | 32133 | NA | 0.01635 | benign | Tolerated | Neutral | Uncertain significance |
|  |  |  | LYST | Het |  |  | 0.3133 | NA | NA | NA | Uncertain significance |
| 70 | 69 | Male | LYST | Het | NA | NA | NA | benign | Tolerated | Neutral | Uncertain significance |
|  |  |  | LYST | Het |  |  | 0 | possibly damaging | Damaging | Neutral | Uncertain significance |
| 71 | 31 | Male | LYST | Het | NA | NA | NA | NA | NA | NA | Possibly pathogenic |
|  |  |  | LYST | Het |  |  | 0.08712 | benign | Tolerated | Neutral | Uncertain significance |
| 72 | 57 | Male | LYST | Het | NA | 2 | NA | NA | NA | NA | Uncertain significance |
|  |  |  | LYST | Het |  |  | 0.02719 | benign | Tolerated | Neutral | Uncertain significance |
| 73 | 2 | Male | LYST | Het | NA | 1.5 | NA | benign | Tolerated | Neutral | Uncertain significance |
|  |  |  | LYST | Het |  |  | 0.02718 | benign | Tolerated | Neutral | Uncertain significance |
| 74 | 21 | Female | LYST | Het | NA | NA | 0.2658 | probably damaging | Damaging | Deleterious | Uncertain significance |
|  |  |  | LYST | Het |  |  | NA | probably damaging | Damaging | Deleterious | Uncertain significance |
| 75 | 0.2 | Female | STXBP2 | Het | 39135 | 2.1 | 0 | probably damaging | Damaging | Deleterious | Uncertain significance |
|  |  |  | STXBP2 | Het |  |  | NA | NA | NA | NA | Possibly pathogenic |
| 76 | 0.7 | Male | STXBP2 | Het | NA | 1.6 | NA | probably damaging | Damaging | Deleterious | Uncertain significance |
|  |  |  | STXBP2 | Het |  |  | 0.02181 | probably damaging | Damaging | Deleterious | Possibly pathogenic |
| 77 | 13 | Female | RAB27A | Hom | NA | NA | NA | NA | NA | NA | Uncertain significance |
| 78 | 30 | Female | RAB27A | Het | NA | NA | NA | probably damaging | Damaging | Deleterious | Uncertain significance |
|  |  |  | RAB27A | Het |  |  | 0 | NA | NA | NA | Possibly pathogenic |

*gnomAD v2.1.1 Eastern Asian population frequency. ACMG, American College of Medical Genetics and Genomics; Het, heterozygous; Hom, homozygous; Hemi, hemizygous; NA, not available; Polyphen-2, Polymorphism Phenotype v2; PROVEAN, Protein Variation Effect Analyzer; SIFT, Sorting Intolerant From Tolerant.

Table S3. Patients with single/digenic/polygenic heterozygous variants.

| Patients | Age at testing, year | Sex | Gene | Zygosity | sCD25  pg/ml | CTLs degranulation (CD107a ΔMFI) | Frequency in gnomAD*, % | Polyphen2 | SIFT | PROVEAN | ACMG  classification |
| --- | --- | --- | --- | --- | --- | --- | --- | --- | --- | --- | --- |
| 1 | 1 | Male | UNC13D | Het | NA | 3.5 | 0.005449 | probably damaging | Damaging | Neutral | Uncertain significance |
| 2 | 64 | Male | UNC13D | Het | 79330 | NA | 0.07526 | benign | Damaging | Neutral | Uncertain significance |
| 3 | 35 | Male | UNC13D | Het | NA | NA | 0.02178 | benign | Tolerated | Neutral | Uncertain significance |
| 4 | 61 | Male | UNC13D | Het | NA | NA | 0.02178 | benign | Tolerated | Neutral | Uncertain significance |
| 5 | 3 | Male | UNC13D | Het | NA | NA | 0 | NA | NA | NA | Uncertain significance |
| 6 | 44 | Male | UNC13D | Het | NA | NA | 0.09527 | possibly damaging | Damaging | Deleterious | Uncertain significance |
| 7 | NA | Male | UNC13D | Het | NA | NA | 0.09527 | possibly damaging | Damaging | Deleterious | Uncertain significance |
| 8 | 20 | Female | UNC13D | Het | NA | 2.4 | 0.09527 | possibly damaging | Damaging | Deleterious | Uncertain significance |
| 9 | 36 | Male | UNC13D | Het | NA | NA | 0.09527 | possibly damaging | Damaging | Deleterious | Uncertain significance |
| 10 | 3.2 | Female | UNC13D | Het | 7267 | NA | 0.09527 | possibly damaging | Damaging | Deleterious | Uncertain significance |
| 11 | 29 | Male | UNC13D | Het | 45395 | 3.4 | 0.05013 | possibly damaging | Tolerated | Neutral | Uncertain significance |
| 12 | 2 | Male | UNC13D | Het | NA | NA | 0.05013 | possibly damaging | Tolerated | Neutral | Uncertain significance |
| 13 | 2 | Male | UNC13D | Het | NA | NA | NA | NA | NA | NA | Pathogenic |
| 14 | 27 | Male | UNC13D | Het | 42437 | NA | 0.09539 | benign | Damaging | Neutral | Uncertain significance |
| 15 | 55 | Male | UNC13D | Het | NA | NA | 0.01505 | benign | Tolerated | Deleterious | Uncertain significance |
| 16 | 23 | Female | UNC13D | Het | NA | NA | 0 | NA | NA | NA | Pathogenic |
| 17 | 2.1 | Male | UNC13D | Het | NA | 3.1 | 0.01003 | possibly damaging | Damaging | Deleterious | Uncertain significance |
| 18 | 2 | Male | UNC13D | Het | NA | NA | 0.05565 | NA | NA | NA | Uncertain significance |
| 19 | 52 | Female | UNC13D | Het | 4324 | NA | 0.05565 | NA | NA | NA | Uncertain significance |
| 20 | 67 | Female | UNC13D | Het | NA | NA | 0.005469 | possibly damaging | Damaging | Neutral | Uncertain significance |
| 21 | 2 | Female | UNC13D | Het | NA | NA | 0 | possibly damaging | Damaging | Deleterious | Uncertain significance |
| 22 | 47 | Female | UNC13D | Het | NA | NA | NA | probably damaging | Damaging | Deleterious | Uncertain significance |
| 23 | 53 | Male | UNC13D | Het | NA | NA | NA | probably damaging | Damaging | Deleterious | Uncertain significance |
| 24 | 0.2 | Male | UNC13D | Het | NA | NA | NA | NA | NA | NA | Pathogenic |
| 25 | 23 | Female | UNC13D | Het | NA | NA | NA | benign | Tolerated | Neutral | Uncertain significance |
| 26 | 45 | Male | UNC13D | Het | 3737 | NA | 1.609 | benign | Tolerated | Neutral | Uncertain significance |
| 27 | 66 | Male | UNC13D | Het | 25720 | 1.1 | 1.609 | benign | Tolerated | Neutral | Uncertain significance |
| 28 | 23 | Female | UNC13D | Het | 28314 | NA | 1.609 | benign | Tolerated | Neutral | Uncertain significance |
| 29 | 26 | Male | UNC13D | Het | 10743 | NA | 1.609 | benign | Tolerated | Neutral | Uncertain significance |
| 30 | 10 | Female | UNC13D | Het | NA | NA | 0.8222 | benign | Tolerated | Neutral | Uncertain significance |
| 31 | 3 | Male | UNC13D | Het | NA | 2.9 | 0 | probably damaging | Damaging | Deleterious | Uncertain significance |
| 32 | 5 | Female | UNC13D | Het | NA | NA | 0.02507 | possibly damaging | Damaging | Deleterious | Uncertain significance |
| 33 | 13 | Female | UNC13D | Het | NA | NA | NA | probably damaging | Damaging | Deleterious | Uncertain significance |
| 34 | 33 | Female | UNC13D | Het | NA | 2.5 | NA | NA | NA | NA | Pathogenic |
| 35 | 60 | Female | UNC13D | Het | NA | NA | NA | benign | Tolerated | Neutral | Uncertain significance |
| 36 | 20 | Male | UNC13D | Het | 40583 | NA | 0 | benign | Tolerated | Neutral | Uncertain significance |
| 37 | 38 | Female | UNC13D | Het | NA | NA | 0.05336 | benign | Tolerated | Neutral | Uncertain significance |
| 38 | 24 | Male | UNC13D | Het | NA | NA | 0.05336 | benign | Tolerated | Neutral | Uncertain significance |
| 39 | NA | Female | UNC13D | Het | NA | NA | 0.05336 | benign | Tolerated | Neutral | Uncertain significance |
| 40 | 3.5 | Male | UNC13D | Het | 2773 | 6.1 | 0.05336 | benign | Tolerated | Neutral | Uncertain significance |
| 41 | 31 | Male | UNC13D | Het | 70315 | NA | NA | benign | Tolerated | Neutral | Uncertain significance |
| 42 | 16 | Male | UNC13D | Het | NA | NA | 0.005017 | benign | Tolerated | Neutral | Uncertain significance |
| 43 | 30 | Male | UNC13D | Het | NA | NA | 0.371 | probably damaging | Damaging | Deleterious | Possibly pathogenic |
| 44 | 0.8 | Male | UNC13D | Het | 79050 | NA | 0.371 | probably damaging | Damaging | Deleterious | Possibly pathogenic |
| 45 | 24 | Male | UNC13D | Het | 118370 | 3.7 | 0.371 | probably damaging | Damaging | Deleterious | Possibly pathogenic |
| 46 | 4 | Male | UNC13D | Het | NA | NA | 0.371 | probably damaging | Damaging | Deleterious | Possibly pathogenic |
| 47 | 2.1 | Female | UNC13D | Het | NA | NA | 0.371 | probably damaging | Damaging | Deleterious | Possibly pathogenic |
| 48 | 40 | Female | UNC13D | Het | NA | NA | 0.371 | probably damaging | Damaging | Deleterious | Possibly pathogenic |
| 49 | 25 | Female | UNC13D | Het | NA | NA | 0.371 | probably damaging | Damaging | Deleterious | Possibly pathogenic |
| 50 | 7 | Female | UNC13D | Het | 10917 | NA | 0.371 | probably damaging | Damaging | Deleterious | Possibly pathogenic |
| 51 | 28 | Male | UNC13D | Het | NA | NA | 0.371 | probably damaging | Damaging | Deleterious | Possibly pathogenic |
| 52 | 29 | Male | UNC13D | Het | NA | NA | 0.371 | probably damaging | Damaging | Deleterious | Possibly pathogenic |
| 53 | 5 | Female | UNC13D | Het | 33123 | NA | 0.371 | probably damaging | Damaging | Deleterious | Possibly pathogenic |
| 54 | 8.1 | Female | UNC13D | Het | NA | NA | 0.371 | probably damaging | Damaging | Deleterious | Possibly pathogenic |
| 55 | 66 | Female | UNC13D | Het | NA | NA | 0.371 | probably damaging | Damaging | Deleterious | Possibly pathogenic |
| 56 | 55 | Male | UNC13D | Het | NA | NA | 0.371 | probably damaging | Damaging | Deleterious | Possibly pathogenic |
| 57 | 24 | Female | UNC13D | Het | NA | NA | 0.371 | probably damaging | Damaging | Deleterious | Possibly pathogenic |
| 58 | 7.1 | Female | UNC13D | Het | 29096 | NA | 0.371 | probably damaging | Damaging | Deleterious | Possibly pathogenic |
| 59 | 38 | Female | UNC13D | Het | 20445 | NA | 0.371 | probably damaging | Damaging | Deleterious | Possibly pathogenic |
| 60 | 24 | Male | UNC13D | Het | NA | NA | 0.371 | probably damaging | Damaging | Deleterious | Possibly pathogenic |
| 61 | 54 | Male | UNC13D | Het | NA | NA | 0.371 | probably damaging | Damaging | Deleterious | Possibly pathogenic |
| 62 | 26 | Female | UNC13D | Het | NA | NA | 0.371 | probably damaging | Damaging | Deleterious | Possibly pathogenic |
| 63 | 34 | Female | UNC13D | Het | NA | NA | 0.371 | probably damaging | Damaging | Deleterious | Possibly pathogenic |
| 64 | 29 | Female | UNC13D | Het | NA | NA | 0.371 | probably damaging | Damaging | Deleterious | Possibly pathogenic |
| 65 | 24 | Male | UNC13D | Het | 877 | NA | NA | probably damaging | Damaging | Deleterious | Uncertain significance |
| 66 | 2 | Male | UNC13D | Het | NA | 2.1 | NA | NA | NA | NA | Possibly pathogenic |
| 67 | 31 | Male | UNC13D | Het | 11308 | NA | NA | benign | Tolerated | Neutral | Uncertain significance |
| 68 | 76 | Male | UNC13D | Het | NA | NA | 0.01008 | benign | Tolerated | Neutral | Uncertain significance |
| 69 | 43 | Female | UNC13D | Het | NA | NA | 0.04717 | possibly damaging | Tolerated | Neutral | Uncertain significance |
| 70 | 54 | Male | UNC13D | Het | 60650 | NA | 0.04717 | possibly damaging | Tolerated | Neutral | Uncertain significance |
| 71 | 3.2 | Male | UNC13D | Het | 29635 | 1.1 | 0.006285 | probably damaging | Damaging | Deleterious | Uncertain significance |
| 72 | 61 | Female | UNC13D | Het | NA | NA | 0 | possibly damaging | Damaging | Deleterious | Uncertain significance |
| 73 | 68 | Female | UNC13D | Het | 34146 | 3.2 | 0 | benign | Tolerated | Neutral | Uncertain significance |
| 74 | 6 | Female | UNC13D | Het | NA | NA | NA | probably damaging | Damaging | Deleterious | Uncertain significance |
| 75 | 7 | Male | UNC13D | Het | NA | NA | 0.1025 | benign | Tolerated | Neutral | Uncertain significance |
| 76 | 40 | Male | UNC13D | Het | NA | NA | 0.02583 | probably damaging | Damaging | Deleterious | Uncertain significance |
| 77 | 26 | Male | UNC13D | Het | NA | NA | 0.02583 | probably damaging | Damaging | Deleterious | Uncertain significance |
| 78 | 9 | Female | UNC13D | Het | 7745 | 2.1 | NA | NA | NA | NA | Uncertain significance |
| 79 | 47 | Male | UNC13D | Het | NA | NA | NA | possibly damaging | Tolerated | Neutral | Uncertain significance |
| 80 | 4.2 | Female | LYST | Het | NA | 6.6 | NA | probably damaging | Damaging | Neutral | Uncertain significance |
| 81 | 28 | Male | LYST | Het | NA | NA | NA | probably damaging | Damaging | Neutral | Uncertain significance |
| 82 | 1 | Female | LYST | Het | NA | NA | 1.147 | benign | Tolerated | Neutral | Uncertain significance |
| 83 | 4 | Female | LYST | Het | 48490 | 2 | 1.147 | benign | Tolerated | Neutral | Uncertain significance |
| 84 | 4 | Male | LYST | Het | 35925 | 3.3 | 1.147 | benign | Tolerated | Neutral | Uncertain significance |
| 85 | 26 | Female | LYST | Het | NA | NA | 1.147 | benign | Tolerated | Neutral | Uncertain significance |
| 86 | 17 | Male | LYST | Het | 272880 | NA | NA | benign | Damaging | Neutral | Uncertain significance |
| 87 | 18 | Male | LYST | Het | NA | NA | 0.01094 | benign | Tolerated | Deleterious | Uncertain significance |
| 88 | 41 | Female | LYST | Het | NA | NA | 0.01094 | benign | Tolerated | Deleterious | Uncertain significance |
| 89 | 0.2 | Male | LYST | Het | NA | NA | NA | probably damaging | Damaging | Deleterious | Uncertain significance |
| 90 | 27 | Female | LYST | Het | NA | NA | 0.005461 | probably damaging | Tolerated | Deleterious | Uncertain significance |
| 91 | 75 | Female | LYST | Het | 99815 | NA | NA | probably damaging | Tolerated | Neutral | Uncertain significance |
| 92 | 54 | Male | LYST | Het | 205870 | NA | 0 | probably damaging | Damaging | Deleterious | Uncertain significance |
| 93 | 49 | Female | LYST | Het | 94200 | NA | NA | probably damaging | Damaging | Neutral | Uncertain significance |
| 94 | 68 | Male | LYST | Het | 31836 | NA | 0.1006 | benign | Tolerated | Neutral | Uncertain significance |
| 95 | 0.4 | Female | LYST | Het | 97050 | NA | 0.01633 | benign | Tolerated | Neutral | Uncertain significance |
| 96 | 7 | Male | LYST | Het | 6428 | NA | NA | benign | Tolerated | Deleterious | Uncertain significance |
| 97 | NA | Female | LYST | Het | 37575 | 2.8 | 0.01635 | benign | Tolerated | Neutral | Uncertain significance |
| 98 | 21 | Male | LYST | Het | 35926 | NA | NA | probably damaging | Tolerated | Neutral | Uncertain significance |
| 99 | 25 | Female | LYST | Het | NA | NA | NA | benign | Damaging | Neutral | Uncertain significance |
| 100 | 39 | Male | LYST | Het | 247520 | NA | 0.02011 | benign | Tolerated | Neutral | Uncertain significance |
| 101 | 1.2 | Male | LYST | Het | NA | NA | 0.02011 | benign | Tolerated | Neutral | Uncertain significance |
| 102 | 2 | Male | LYST | Het | 6410 | NA | NA | benign | Tolerated | Neutral | Uncertain significance |
| 103 | NA | Male | LYST | Het | NA | NA | 0.03274 | benign | Damaging | Deleterious | Uncertain significance |
| 104 | 13 | Male | LYST | Het | NA | NA | 0.02177 | benign | Tolerated | Neutral | Uncertain significance |
| 105 | 30 | Male | LYST | Het | NA | 2.4 | 0.01006 | probably damaging | Tolerated | Deleterious | Uncertain significance |
| 106 | 3 | Male | LYST | Het | NA | 2.8 | 0 | benign | Tolerated | Deleterious | Uncertain significance |
| 107 | 51 | Female | LYST | Het | NA | NA | 0.005445 | benign | Tolerated | Deleterious | Uncertain significance |
| 108 | 24 | Male | LYST | Het | NA | 2.3 | NA | benign | Tolerated | Neutral | Uncertain significance |
| 109 | 65 | Female | LYST | Het | 3251 | NA | NA | NA | NA | NA | Uncertain significance |
| 110 | 55 | Male | LYST | Het | 24444 | NA | NA | possibly damaging | Damaging | Neutral | Uncertain significance |
| 111 | 40 | Male | LYST | Het | NA | NA | NA | benign | Damaging | Neutral | Uncertain significance |
| 112 | 10 | Female | LYST | Het | NA | NA | NA | benign | Damaging | Neutral | Uncertain significance |
| 113 | 37 | Male | LYST | Het | NA | 5.9 | NA | benign | Damaging | Neutral | Uncertain significance |
| 114 | 2.9 | Male | LYST | Het | 1478 | 3.5 | 0 | benign | Tolerated | Neutral | Uncertain significance |
| 115 | 38 | Male | LYST | Het | NA | 4 | 0 | benign | Tolerated | Neutral | Uncertain significance |
| 116 | 1.2 | Male | LYST | Het | 176795 | 4.2 | 0.02176 | probably damaging | Damaging | Deleterious | Uncertain significance |
| 117 | 2 | Male | LYST | Het | 42964 | NA | 0.02176 | probably damaging | Damaging | Deleterious | Uncertain significance |
| 118 | 70 | Male | LYST | Het | 40774 | 3.9 | 0.005447 | probably damaging | Tolerated | Deleterious | Uncertain significance |
| 119 | 6 | Female | LYST | Het | 10559 | NA | 0.005447 | probably damaging | Tolerated | Deleterious | Uncertain significance |
| 120 | 6 | Female | LYST | Het | 10982 | NA | NA | benign | Tolerated | Neutral | Uncertain significance |
| 121 | 1 | Female | LYST | Het | NA | 2.1 | 0.02719 | benign | Tolerated | Neutral | Uncertain significance |
| 122 | 75 | Female | LYST | Het | 25128 | NA | 0 | benign | Tolerated | Neutral | Uncertain significance |
| 123 | 15 | Male | LYST | Het | NA | NA | 0 | probably damaging | Damaging | Neutral | Uncertain significance |
| 124 | 29 | Female | LYST | Het | NA | NA | NA | benign | Tolerated | Neutral | Uncertain significance |
| 125 | 10.6 | Male | LYST | Het | NA | 2.3 | 0.02175 | benign | Tolerated | Neutral | Uncertain significance |
| 126 | 67 | Male | LYST | Het | NA | NA | 0.02175 | benign | Tolerated | Neutral | Uncertain significance |
| 127 | 51 | Male | LYST | Het | 35253 | NA | 0 | benign | Damaging | Neutral | Uncertain significance |
| 128 | 51 | Male | LYST | Het | NA | NA | NA | benign | Tolerated | Neutral | Uncertain significance |
| 129 | 22 | Female | LYST | Het | NA | NA | 0.01632 | benign | Damaging | Deleterious | Uncertain significance |
| 130 | 12 | Male | LYST | Het | NA | 3.1 | NA | benign | Tolerated | Neutral | Uncertain significance |
| 131 | 6.6 | Female | LYST | Het | 35098 | NA | 0.02718 | benign | Tolerated | Neutral | Uncertain significance |
| 132 | 0.3 | Female | LYST | Het | NA | NA | 0.01631 | benign | Tolerated | Neutral | Uncertain significance |
| 133 | 32 | Female | LYST | Het | 9769 | NA | 0.02005 | probably damaging | Damaging | Deleterious | Uncertain significance |
| 134 | 1.2 | Male | LYST | Het | NA | NA | 0 | probably damaging | Damaging | Deleterious | Uncertain significance |
| 135 | 35 | Female | LYST | Het | NA | NA | 0 | probably damaging | Damaging | Deleterious | Uncertain significance |
| 136 | 68 | Male | LYST | Het | NA | NA | 0.01504 | probably damaging | Damaging | Deleterious | Uncertain significance |
| 137 | 56 | Male | LYST | Het | 100260 | NA | 0.01631 | benign | Tolerated | Neutral | Uncertain significance |
| 138 | 32 | Female | LYST | Het | 9439 | NA | 0.005437 | benign | Tolerated | Neutral | Uncertain significance |
| 139 | 34 | Female | LYST | Het | NA | NA | 0.005437 | possibly damaging | Tolerated | Neutral | Uncertain significance |
| 140 | 50 | Male | LYST | Het | 29380 | NA | NA | benign | Tolerated | Neutral | Uncertain significance |
| 141 | 18 | Female | LYST | Het | NA | NA | NA | benign | Tolerated | Neutral | Uncertain significance |
| 142 | NA | Male | LYST | Het | 100830 | NA | 0.03263 | probably damaging | Tolerated | Neutral | Uncertain significance |
| 143 | 3.2 | Female | PRF1 | Het | 23320 | NA | 0.6853 | benign | Damaging | Neutral | Uncertain significance |
| 144 | 11 | Female | PRF1 | Het | NA | NA | 0 | probably damaging | Damaging | Neutral | Pathogenic |
| 145 | 10 | Female | PRF1 | Het | NA | NA | 0 | probably damaging | Damaging | Deleterious | Uncertain significance |
| 146 | 4 | Female | PRF1 | Het | 22060 | 4 | 0.06562 | benign | Damaging | Neutral | Uncertain significance |
| 147 | 2 | Female | PRF1 | Het | NA | 3.3 | 0.005024 | benign | Tolerated | Deleterious | Uncertain significance |
| 148 | 4 | Male | PRF1 | Het | 6715 | NA | 0 | benign | Tolerated | Neutral | Uncertain significance |
| 149 | 1 | Female | PRF1 | Het | 34344 | NA | 0.01506 | possibly damaging | Damaging | Deleterious | Uncertain significance |
| 150 | 51 | Male | PRF1 | Het | NA | NA | 0 | probably damaging | Damaging | Deleterious | Uncertain significance |
| 151 | 1.5 | Male | PRF1 | Het | 6475 | NA | 0.05012 | benign | Tolerated | Neutral | Possibly pathogenic |
| 152 | 1.8 | Male | PRF1 | Het | NA | NA | 0.05012 | benign | Tolerated | Neutral | Possibly pathogenic |
| 153 | 8 | Female | PRF1 | Het | NA | NA | 0.005439 | possibly damaging | Damaging | Deleterious | Pathogenic |
| 154 | 1 | Female | PRF1 | Het | 24435 | NA | 0.04512 | benign | Tolerated | Neutral | Uncertain significance |
| 155 | 52 | Male | PRF1 | Het | 14815 | NA | NA | benign | Tolerated | Neutral | Pathogenic |
| 156 | 4 | Female | PRF1 | Het | NA | 2.8 | 0 | possibly damaging | Damaging | Deleterious | Uncertain significance |
| 157 | 1.7 | Male | PRF1 | Het | 31531 | 2.3 | 0 | possibly damaging | Damaging | Deleterious | Uncertain significance |
| 158 | 15 | Male | PRF1 | Het | 37213 | NA | 0.08024 | probably damaging | Tolerated | Deleterious | Uncertain significance |
| 159 | 30 | Female | PRF1 | Het | 37888 | NA | 0 | NA | NA | NA | Pathogenic |
| 160 | 43 | Male | PRF1 | Het | NA | NA | 0.03519 | possibly damaging | Damaging | Deleterious | Pathogenic |
| 161 | 69 | Female | PRF1 | Het | 133155 | NA | 0.0109 | benign | Tolerated | Deleterious | Uncertain significance |
| 162 | 1.2 | Female | PRF1 | Het | NA | NA | NA | possibly damaging | Tolerated | Neutral | Uncertain significance |
| 163 | 17 | Male | PRF1 | Het | NA | NA | 0.005438 | probably damaging | Damaging | Deleterious | Uncertain significance |
| 164 | 5.2 | Male | PRF1 | Het | NA | NA | 0.005437 | possibly damaging | Damaging | Deleterious | Pathogenic |
| 165 | 35 | Female | PRF1 | Het | 3092 | NA | 0.005437 | possibly damaging | Damaging | Deleterious | Pathogenic |
| 166 | 1.4 | Female | PRF1 | Het | NA | NA | 0.005437 | possibly damaging | Damaging | Deleterious | Pathogenic |
| 167 | 29 | Female | PRF1 | Het | NA | NA | 0.005437 | possibly damaging | Damaging | Deleterious | Pathogenic |
| 168 | 51 | Female | PRF1 | Het | 22199 | NA | 0 | benign | Tolerated | Neutral | Uncertain significance |
| 169 | 57 | Male | PRF1 | Het | NA | NA | NA | NA | NA | NA | Possibly pathogenic |
| 170 | NA | Female | PRF1 | Het | 27255 | 2.9 | NA | possibly damaging | Damaging | Deleterious | Uncertain significance |
| 171 | 55 | Female | STXBP2 | Het | 11854 | NA | NA | benign | Tolerated | Neutral | Uncertain significance |
| 172 | 11 | Female | STXBP2 | Het | 4832 | 4.9 | 0.03806 | possibly damaging | Tolerated | Neutral | Uncertain significance |
| 173 | 3 | Female | STXBP2 | Het | 40511 | 5.3 | NA | benign | Tolerated | Neutral | Uncertain significance |
| 174 | 3.3 | Male | STXBP2 | Het | 4040 | NA | NA | probably damaging | Damaging | Deleterious | Uncertain significance |
| 175 | 0.8 | Male | STXBP2 | Het | 27636 | NA | NA | probably damaging | Tolerated | Deleterious | Uncertain significance |
| 176 | 1.8 | Male | STXBP2 | Het | 171555 | 2.3 | NA | probably damaging | Damaging | Deleterious | Uncertain significance |
| 177 | 4 | Male | STXBP2 | Het | NA | NA | 0.03492 | probably damaging | Damaging | Deleterious | Uncertain significance |
| 178 | 0.8 | Female | STXBP2 | Het | 1204 | 3.7 | 0.03492 | probably damaging | Damaging | Deleterious | Uncertain significance |
| 179 | 1.1 | Female | STXBP2 | Het | 13670 | NA | 0.02007 | benign | Tolerated | Neutral | Uncertain significance |
| 180 | 2.2 | Female | STXBP2 | Het | NA | NA | NA | probably damaging | Damaging | Deleterious | Uncertain significance |
| 181 | 1.6 | Male | STXBP2 | Het | NA | 1.9 | NA | possibly damaging | Damaging | Deleterious | Uncertain significance |
| 182 | 3.7 | Female | STXBP2 | Het | NA | NA | 0.5014 | probably damaging | Tolerated | Neutral | Uncertain significance |
| 183 | 46 | Female | STXBP2 | Het | NA | NA | 0.005437 | benign | Tolerated | Neutral | Uncertain significance |
| 184 | 1 | Male | STXBP2 | Het | NA | NA | 0.02178 | possibly damaging | Tolerated | Neutral | Uncertain significance |
| 185 | 68 | Male | STXBP2 | Het | 78770 | 4.2 | NA | NA | NA | NA | Possibly pathogenic |
| 186 | 19 | Male | STXBP2 | Het | NA | NA | NA | benign | Damaging | Neutral | Uncertain significance |
| 187 | 1 | Male | STXBP2 | Het | 10795 | NA | 0.04425 | NA | NA | NA | Uncertain significance |
| 188 | 14 | Male | STXBP2 | Het | 34935 | NA | 0.04425 | NA | NA | NA | Uncertain significance |
| 189 | 54 | Male | STXBP2 | Het | 78770 | 2.8 | 0.005121 | probably damaging | Damaging | Deleterious | Uncertain significance |
| 190 | 27 | Male | STXBP2 | Het | NA | NA | 0.005121 | probably damaging | Damaging | Deleterious | Uncertain significance |
| 191 | NA | Female | STXBP2 | Het | 1332 | NA | NA | possibly damaging | Damaging | Deleterious | Uncertain significance |
| 192 | 40 | Male | STXBP2 | Het | NA | NA | 0.005158 | probably damaging | Damaging | Deleterious | Uncertain significance |
| 193 | 11 | Male | STXBP2 | Het | NA | 4.1 | NA | benign | Tolerated | Neutral | Uncertain significance |
| 194 | 9 | Male | AP3B1 | Het | 14648 | NA | 0.00544 | benign | Tolerated | Neutral | Uncertain significance |
| 195 | 46 | Female | AP3B1 | Het | NA | NA | NA | NA | NA | NA | Uncertain significance |
| 196 | 7 | Male | AP3B1 | Het | NA | 1 | NA | possibly damaging | Damaging | Deleterious | Uncertain significance |
| 197 | 32 | Male | AP3B1 | Het | 12377 | NA | NA | probably damaging | Damaging | Deleterious | Uncertain significance |
| 198 | 41 | Female | AP3B1 | Het | NA | NA | NA | benign | Tolerated | Deleterious | Uncertain significance |
| 199 | 23 | Male | AP3B1 | Het | NA | NA | NA | benign | Damaging | Deleterious | Uncertain significance |
| 200 | 4 | Male | AP3B1 | Het | 33502 | NA | 0.03508 | possibly damaging | Damaging | Deleterious | Uncertain significance |
| 201 | 62 | Male | AP3B1 | Het | NA | NA | NA | NA | NA | NA | Possibly pathogenic |
| 202 | 5 | Female | AP3B1 | Het | 23268 | NA | NA | benign | Tolerated | Neutral | Uncertain significance |
| 203 | 4 | Male | AP3B1 | Het | 23174 | NA | NA | benign | Damaging | Deleterious | Uncertain significance |
| 204 | 13 | Male | AP3B1 | Het | 33387 | NA | 0 | benign | Tolerated | Neutral | Uncertain significance |
| 205 | 64 | Female | AP3B1 | Het | 38946 | NA | 0.09253 | benign | Damaging | Deleterious | Uncertain significance |
| 206 | 1.4 | Female | AP3B1 | Het | 2652 | 1.8 | 0.1002 | benign | Damaging | Deleterious | Uncertain significance |
| 207 | 35 | Male | AP3B1 | Het | 158300 | NA | 0.1002 | benign | Damaging | Deleterious | Uncertain significance |
| 208 | 23 | Female | STX11 | Het | NA | NA | 0.02721 | benign | Tolerated | Neutral | Uncertain significance |
| 209 | 47 | Male | STX11 | Het | NA | 2.8 | NA | benign | Tolerated | Neutral | Uncertain significance |
| 210 | 1.2 | Male | STX11 | Het | 7803 | NA | NA | benign | Damaging | Neutral | Uncertain significance |
| 211 | 38 | Male | STX11 | Het | 145660 | NA | 0.02182 | benign | Tolerated | Neutral | Uncertain significance |
| 212 | 5 | Female | STX11 | Het | NA | NA | NA | probably damaging | Damaging | Deleterious | Uncertain significance |
| 213 | 4 | Female | STX11 | Het | 174760 | NA | NA | benign | Damaging | Neutral | Uncertain significance |
| 214 | 47 | Male | STX11 | Het | NA | NA | NA | probably damaging | Tolerated | Neutral | Uncertain significance |
| 215 | 30 | Female | STX11 | Het | NA | NA | 0 | possibly damaging | Tolerated | Neutral | Uncertain significance |
| 216 | 7 | Male | RAB27A | Het | NA | 1.5 | 0.005439 | probably damaging | Damaging | Deleterious | Uncertain significance |
| 217 | 1 | Male | RAB27A | Het | NA | 3 | 0.0218 | probably damaging | Damaging | Deleterious | Uncertain significance |
| 218 | 19 | Female | RAB27A | Het | NA | 1.2 | 0.6866 | benign | Tolerated | Neutral | Uncertain significance |
| 219 | 19 | Female | RAB27A | Het | 4156 | NA | 0.6866 | benign | Tolerated | Neutral | Uncertain significance |
| 220 | 23 | Male | RAB27A | Het | 4240 | NA | 0.6866 | benign | Tolerated | Neutral | Uncertain significance |
| 221 | 16 | Female | RAB27A | Het | NA | NA | NA | benign | Tolerated | Neutral | Uncertain significance |
| 222 | 3 | Male | UNC13D | Het | NA | 1.5 | 1.609 | benign | Tolerated | Neutral | Uncertain significance |
|  |  |  | LYST | Het | NA | NA | 0.1757 | benign | Damaging | Deleterious | Uncertain significance |
| 223 | 5 | Female | UNC13D | Het | 1805 | 3.1 | 0.371 | probably damaging | Damaging | Deleterious | Possibly pathogenic |
|  |  |  | LYST | Het | NA | NA | 0.1757 | benign | Damaging | Deleterious | Uncertain significance |
| 224 | 75 | Female | UNC13D | Het | 33272 | NA | 0.02245 | possibly damaging | Tolerated | Deleterious | Uncertain significance |
|  |  |  | LYST | Het | NA | NA | 0.06019 | benign | Damaging | Neutral | Uncertain significance |
| 225 | 0.3 | Female | UNC13D | Het | 191910 | 2.8 | 1.609 | benign | Tolerated | Neutral | Uncertain significance |
|  |  |  | LYST | Het | NA | NA | NA | probably damaging | Damaging | Deleterious | Uncertain significance |
| 226 | 8.1 | Female | UNC13D | Het | 18420 | NA | 1.609 | benign | Tolerated | Neutral | Uncertain significance |
|  |  |  | LYST | Het | NA | NA | 1.147 | benign | Tolerated | Neutral | Uncertain significance |
|  |  |  | RAB27A | Het | NA | NA | 0.6866 | benign | Tolerated | Neutral | Uncertain significance |
| 227 | 58 | Female | UNC13D | Het | NA | 2.3 | 0.04717 | possibly damaging | Tolerated | Neutral | Uncertain significance |
|  |  |  | AP3B1 | Het | NA | NA | NA | NA | NA | NA | Possibly pathogenic |
| 228 | 1 | Female | UNC13D | Het | 15815 | 2.1 | NA | NA | NA | NA | Pathogenic |
|  |  |  | AP3B1 | Het | NA | NA | NA | benign | Damaging | Deleterious | Uncertain significance |
| 229 | 17 | Female | UNC13D | Het | 37382 | 2 | 0.01632 | probably damaging | Tolerated | Neutral | Uncertain significance |
|  |  |  | RAB27A | Het | NA | NA | NA | NA | NA | NA | Possibly pathogenic |
| 230 | 26 | Female | UNC13D | Het | 24947 | 2.4 | 0.005441 | possibly damaging | Damaging | Deleterious | Uncertain significance |
|  |  |  | RAB27A | Het | NA | NA | 0.03838 | benign | Tolerated | Neutral | Uncertain significance |
| 231 | 2.1 | Female | LYST | Het | 38120 | 2.2 | 0.08712 | benign | Tolerated | Neutral | Uncertain significance |
|  |  |  | STXBP2 | Het | NA | NA | 0.005475 | probably damaging | Damaging | Deleterious | Uncertain significance |
| 232 | 19 | Female | LYST | Het | 33078 | 1.2 | NA | possibly damaging | Damaging | Neutral | Uncertain significance |
|  |  |  | RAB27A | Het | NA | NA | 0.6866 | benign | Tolerated | Neutral | Uncertain significance |
| 233 | 57 | Male | LYST | Het | NA | 2 | 0.01632 | benign | Damaging | Deleterious | Uncertain significance |
|  |  |  | RAB27A | Het | NA | NA | 0.005012 | benign | Damaging | Deleterious | Uncertain significance |
| 234 | 1 | Female | LYST | Het | 39738 | 1.2 | NA | benign | Tolerated | Neutral | Uncertain significance |
|  |  |  | STXBP2 | Het | NA | NA | 0 | probably damaging | Tolerated | Deleterious | Uncertain significance |
| 235 | 37 | Male | LYST | Het | NA | 1.9 | NA | possibly damaging | Damaging | Deleterious | Uncertain significance |
|  |  |  | STXBP2 | Het | NA | NA | 0.02181 | probably damaging | Damaging | Deleterious | Possibly pathogenic |
| 236 | 48 | Female | LYST | Het | NA | 2.1 | 0 | benign | Damaging | Neutral | Uncertain significance |
|  |  |  | STX11 | Het | NA | NA | 0 | benign | Damaging | Neutral | Uncertain significance |

*gnomAD v2.1.1 Eastern Asian population frequency. ACMG, American College of Medical Genetics and Genomics; Het, heterozygous; Hom, homozygous; Hemi, hemizygous; NA, not available; Polyphen-2, Polymorphism Phenotype v2; PROVEAN, Protein Variation Effect Analyzer; SIFT, Sorting Intolerant From Tolerant.

Table S4 Patients without variants in cytotoxic pathway genes.

| Patients | Age at testing, year | Sex | sCD25  pg/ml | CTLs degranulation (CD107a ΔMFI) |
| --- | --- | --- | --- | --- |
| 1 | 2 | Male | 39552 | 3.4 |
| 2 | 23 | Female | 80355 | NA |
| 3 | 3 | Female | 7194 | NA |
| 4 | 37 | Male | 37350 | NA |
| 5 | 2 | Male | 8661 | NA |
| 6 | 21 | Female | 9542 | NA |
| 7 | 21 | Male | 241030 | NA |
| 8 | 26 | Male | 17957 | NA |
| 9 | 52 | Male | 28100 | NA |
| 10 | 4.2 | Female | 2165 | NA |
| 11 | 7 | Male | 34514 | NA |
| 12 | 14 | Male | 38424 | 5.7 |
| 13 | 15 | Male | 24224 | NA |
| 14 | 0.3 | Male | 28226 | NA |
| 15 | 1.7 | Male | NA | 1.8 |
| 16 | NA | Male | 148635 | NA |
| 17 | 31 | Male | 14868 | NA |
| 18 | 31 | Male | 31213 | NA |
| 19 | 24 | Female | 7148 | NA |
| 20 | 52 | Female | 6441 | NA |
| 21 | 32 | Female | 24901 | NA |
| 22 | 28 | Male | 13693 | NA |
| 23 | 5.6 | Female | 169210 | NA |
| 24 | 28 | Male | 30793 | NA |
| 25 | 1.5 | Female | NA | 2.9 |
| 26 | 13 | Male | 4662 | NA |
| 27 | 71 | Male | 189095 | NA |
| 28 | 3 | Male | NA | NA |
| 29 | 31 | Male | NA | 2.2 |
| 30 | 2.6 | Male | 24533 | NA |
| 31 | 12 | Male | 3716 | NA |
| 32 | 38 | Male | NA | NA |
| 33 | 4.8 | Male | NA | 3.8 |
| 34 | 10 | Male | NA | 2 |
| 35 | 4.4 | Male | NA | 5.2 |
| 36 | 21 | Male | NA | NA |
| 37 | 9 | Female | 37045 | NA |
| 38 | 8 | Female | 21611 | NA |
| 39 | 65 | Male | 35384 | NA |
| 40 | 64 | Female | 33152 | 1.1 |
| 41 | 6 | Female | 43699 | NA |
| 42 | 37 | Female | 26287 | NA |
| 43 | 6.2 | Male | NA | NA |
| 44 | 3 | Female | NA | 3.1 |
| 45 | 31 | Male | 231275 | NA |
| 46 | 5 | Male | 7258 | NA |
| 47 | 33 | Female | 3573 | NA |
| 48 | 13.7 | Male | 36805 | 3.3 |
| 49 | 34 | Female | 5167 | 1.7 |
| 50 | 1 | Female | 75705 | NA |
| 51 | 34 | Female | NA | 5.9 |
| 52 | 1.2 | Male | 12399 | 3.2 |
| 53 | 24 | Male | 5840 | NA |
| 54 | 1 | Male | NA | 3.2 |
| 55 | 29 | Female | 26699 | NA |
| 56 | 29 | Male | 28233 | NA |
| 57 | 79 | Male | 42577 | NA |
| 58 | 2 | Male | NA | 1.5 |
| 59 | 7 | Female | NA | 2 |
| 60 | 7.9 | Female | NA | NA |
| 61 | 65 | Male | 153200 | NA |
| 62 | 55 | Female | 35897 | NA |
| 63 | 8 | Male | NA | NA |
| 64 | 5 | Male | 19201 | 5.3 |
| 65 | 46 | Female | 22914 | NA |
| 66 | 1 | Male | NA | NA |
| 67 | 53 | Male | 199670 | NA |
| 68 | 1.5 | Female | NA | NA |
| 69 | 78 | Male | 29093 | NA |
| 70 | 0.2 | Male | 38770 | NA |
| 71 | 8 | Female | 23629 | NA |
| 72 | 4 | Male | 5269 | NA |
| 73 | 1.6 | Male | 17577 | NA |
| 74 | 4 | Male | NA | 1.3 |
| 75 | 67 | Female | 31727 | NA |
| 76 | 31 | Female | NA | NA |
| 77 | 34 | Female | 7033 | NA |
| 78 | 2 | Female | NA | 3.4 |
| 79 | 63 | Female | 21710 | NA |
| 80 | 6 | Male | 15330 | NA |
| 81 | 26 | Male | 137020 | NA |
| 82 | 3.9 | Female | 3976 | 3 |
| 83 | 42 | Male | 22509 | 2.6 |
| 84 | 11 | Male | NA | NA |
| 85 | 67 | Female | 9277 | NA |
| 86 | 21 | Female | 15158 | NA |
| 87 | 56 | Male | 36877 | NA |
| 88 | 6 | Male | 320810 | NA |
| 89 | 1 | Male | 112210 | NA |
| 90 | 2 | Male | 14443 | NA |
| 91 | 10 | Male | NA | 4.3 |
| 92 | 35 | Male | 30204 | NA |
| 93 | 57 | Male | 8112 | NA |
| 94 | 13 | Male | NA | NA |
| 95 | 56 | Male | 240990 | NA |
| 96 | 0.1 | Female | 4104 | 3 |
| 97 | 3 | Female | NA | 1.2 |
| 98 | 4 | Male | 39406 | 2.5 |
| 99 | 31 | Male | 7308 | NA |
| 100 | 33 | Male | NA | 5 |
| 101 | 2 | Female | NA | NA |
| 102 | 4.7 | Female | 34961 | NA |
| 103 | 7 | Male | 38718 | NA |
| 104 | 58 | Male | NA | NA |
| 105 | 66 | Male | NA | NA |
| 106 | 66 | Male | 2254 | NA |
| 107 | 23 | Female | 132920 | NA |
| 108 | 2 | Male | NA | 3.9 |
| 109 | 1.5 | Female | 5946 | 2.6 |
| 110 | 25 | Female | 26319 | 5.9 |
| 111 | 62 | Female | 35944 | 5.3 |
| 112 | 52 | Female | NA | 2.7 |
| 113 | 1.2 | Male | NA | 7.2 |
| 114 | 49 | Male | NA | 2.9 |
| 115 | 4 | Female | 234045 | 3 |
| 116 | 25 | Male | 42395 | NA |
| 117 | 33 | Male | NA | 2.1 |
| 118 | 2 | Male | 38163 | 1.3 |
| 119 | 26 | Female | 40607 | NA |
| 120 | 0.7 | Female | 4817 | NA |
| 121 | 2 | Male | 150890 | NA |
| 122 | 73 | Female | NA | 1.3 |
| 123 | 58 | Female | 266205 | NA |
| 124 | 21 | Male | 31496 | NA |
| 125 | 1 | Male | 6613 | 1.8 |
| 126 | 30 | Female | 13980 | 3.8 |
| 127 | 8 | Male | 9715 | NA |
| 128 | 59 | Female | NA | NA |
| 129 | 0.3 | Male | NA | NA |
| 130 | 38 | Male | 15503 | NA |
| 131 | 34 | Male | 3068 | NA |
| 132 | 29 | Male | NA | 2.8 |
| 133 | 4 | Female | 14212 | 2.5 |
| 134 | 2.5 | Female | NA | 3 |
| 135 | 67 | Female | 27564 | NA |
| 136 | 7 | Male | 134755 | NA |
| 137 | 33 | Male | 57885 | NA |
| 138 | 2 | Male | NA | 2.1 |
| 139 | 26 | Female | 29052 | NA |
| 140 | 36 | Female | 37354 | NA |
| 141 | 48 | Male | 33365 | NA |
| 142 | 60 | Male | 127600 | NA |
| 143 | 2.5 | Female | 9902 | NA |
| 144 | 67 | Male | 120480 | NA |
| 145 | 38 | Female | 10614 | NA |
| 146 | 65 | Male | NA | 3.5 |
| 147 | 10 | Female | 43444 | NA |
| 148 | 41 | Male | 12245 | NA |
| 149 | 25 | Female | 14236 | NA |
| 150 | 1 | Male | 17381 | NA |
| 151 | 54 | Male | 120760 | NA |
| 152 | 1 | Male | 99305 | 2.5 |
| 153 | NA | Female | 97555 | NA |
| 154 | 16 | Male | 158955 | NA |
| 155 | 2 | Female | NA | 2.6 |
| 156 | 50 | Male | 114150 | NA |
| 157 | NA | Male | 32892 | NA |
| 158 | 3 | Male | 31964 | NA |
| 159 | NA | Female | NA | 4 |
| 160 | 24 | Female | 15508 | NA |
| 161 | 0.8 | Female | 98195 | NA |
| 162 | 9 | Female | 9106 | NA |
| 163 | 40 | Female | 15255 | NA |
| 164 | 0.9 | Male | 15690 | 2 |
| 165 | 15 | Female | 47935 | NA |
| 166 | 65 | Male | 12729 | NA |
| 167 | 45 | Male | 1244 | NA |
| 168 | 6.3 | Male | NA | 2.2 |
| 169 | 71 | Female | 10890 | 1.7 |
| 170 | 50 | Female | NA | NA |
| 171 | 2 | Male | 21836 | NA |
| 172 | 24 | Male | 32396 | NA |
| 173 | 26 | Female | 179945 | NA |
| 174 | 0.8 | Male | NA | 3.4 |
| 175 | 0.8 | Male | NA | NA |
| 176 | 15 | Male | 13512 | NA |
| 177 | 1.6 | Male | NA | 3.6 |
| 178 | 11 | Male | NA | 4.1 |
| 179 | 5 | Female | 25749 | 3.4 |
| 180 | 63 | Male | 131580 | NA |
| 181 | 53 | Female | 33660 | NA |
| 182 | 35 | Male | 25753 | 1.5 |
| 183 | 13 | Male | 1873 | NA |
| 184 | 5.3 | Female | NA | 7.6 |
| 185 | 52 | Female | NA | 1.6 |
| 186 | 4 | Male | NA | 2.3 |
| 187 | 26 | Female | NA | 2.2 |
| 188 | 13 | Female | NA | 2.8 |
| 189 | 53 | Male | NA | 5.9 |
| 190 | 3 | Female | NA | 1.7 |
| 191 | 4 | Male | NA | 3.1 |
| 192 | 25 | Female | NA | 4 |
| 193 | 10.9 | Female | NA | 1.4 |
| 194 | 2 | Female | NA | 3.8 |
| 195 | 5 | Female | NA | 2.1 |
| 196 | 2.9 | Female | NA | 1.1 |
| 197 | 0.8 | Female | NA | 2.4 |
| 198 | 1 | Male | NA | 2.2 |
| 199 | 41 | Female | NA | 2.3 |
| 200 | 2.9 | Male | NA | 1.4 |
| 201 | 60 | Female | NA | 1 |
| 202 | 8 | Male | NA | 2.8 |
| 203 | 2.9 | Male | NA | 1.4 |
| 204 | 2 | Female | NA | 2.5 |
| 205 | 54 | Male | NA | 1.5 |
| 206 | 1.7 | Female | NA | 2 |
| 207 | 17 | Female | NA | 2.5 |
| 208 | 37 | Female | NA | 2 |
| 209 | 1 | Female | NA | 3.5 |
| 210 | 1 | Female | NA | 7.1 |
| 211 | 3 | Female | NA | 3 |
| 212 | 0.1 | Female | NA | 2.6 |
| 213 | 13 | Female | NA | 3.1 |
| 214 | 1.1 | Male | NA | 1.2 |
| 215 | 2 | Female | NA | 2.1 |
| 216 | 3 | Male | NA | 3.1 |
| 217 | 63 | Male | NA | 3.4 |
| 218 | NA | Female | NA | 2.1 |
| 219 | 5 | Male | NA | 4.5 |
| 220 | 9 | Female | NA | 2.7 |
| 221 | 0.8 | Male | NA | 1.5 |
| 222 | 7 | Female | NA | 2.4 |
| 223 | 18 | Male | NA | 1.6 |
| 224 | 9 | Female | NA | 4 |
| 225 | 2 | Female | NA | 2.9 |
| 226 | 22 | Male | NA | 9 |
| 227 | 31 | Female | NA | 4.7 |
| 228 | 1.6 | Female | NA | 2.5 |
| 229 | 5 | Female | NA | 9.1 |
| 230 | 2 | Female | NA | 1.9 |
| 231 | 2.9 | Male | NA | 3.1 |
| 232 | 3.5 | Male | NA | 2.5 |
| 233 | 22 | Female | NA | 3 |
| 234 | 8 | Female | NA | 2.1 |
| 235 | 4 | Male | NA | 2 |
| 236 | 3 | Female | NA | 2.1 |
| 237 | 5 | Female | NA | 9.2 |
| 238 | 7.8 | Male | NA | 1.8 |
| 239 | 3.3 | Male | NA | 1.5 |
| 240 | 1 | Female | NA | 4.3 |
| 241 | 45 | Male | NA | 1.7 |
| 242 | 10 | Female | NA | 2.1 |
| 243 | 7 | Female | NA | 2.5 |
| 244 | 6.5 | Male | NA | 2.5 |
| 245 | 17 | Female | NA | 3 |
| 246 | 9 | Male | NA | 1.8 |
| 247 | 34 | Female | NA | 7.7 |
| 248 | 38 | Male | NA | 2.8 |
| 249 | 8 | Female | NA | 4.2 |
| 250 | 27 | Male | NA | 2 |
| 251 | 3 | Female | NA | 3.9 |
| 252 | 54 | Female | NA | 9.5 |
| 253 | 13 | Female | NA | 2.6 |
| 254 | 25 | Male | NA | 3.3 |
| 255 | 4 | Male | NA | 4.4 |
| 256 | 3 | Male | NA | NA |
| 257 | 1.5 | Male | NA | 1.5 |
| 258 | 6 | Female | NA | 1.9 |
| 259 | NA | Male | NA | 5.6 |
| 260 | 2 | Male | NA | 4.3 |
| 261 | 8 | Female | NA | 3 |
| 262 | 3.1 | Female | NA | 1.7 |
| 263 | 0.5 | Female | NA | 3 |
| 264 | 44 | Female | NA | 2 |
| 265 | 1 | Male | NA | 2.8 |
| 266 | 2.9 | Male | NA | 4.5 |
| 267 | 3.9 | Female | NA | 1.7 |
| 268 | 4 | Male | NA | 2.8 |
| 269 | 27 | Male | NA | 3.2 |
| 270 | 13 | Male | NA | 1.3 |
| 271 | 1.5 | Male | NA | 2.7 |
| 272 | 3 | Male | NA | 1.8 |
| 273 | 8 | Female | NA | 2.4 |
| 274 | 55 | Male | NA | 2.6 |
| 275 | 4 | Female | NA | 2.9 |
| 276 | 2 | Female | NA | 2.3 |
| 277 | 15 | Female | NA | 3.7 |
| 278 | 1.4 | Female | NA | 1.2 |
| 279 | 2.8 | Female | NA | 4.1 |
| 280 | 5 | Female | NA | 1.9 |
| 281 | 1.9 | Female | NA | 2.7 |
| 282 | 3.7 | Female | NA | 3.6 |
| 283 | 5 | Female | NA | 2.2 |
| 284 | 1 | Male | NA | 2.8 |
| 285 | 33 | Male | NA | 2.5 |
| 286 | 5 | Female | NA | 3.6 |
| 287 | 38 | Male | NA | 6.3 |
| 288 | 10 | Female | NA | 2.3 |
| 289 | 53 | Female | NA | 2.7 |
| 290 | 1 | Female | NA | 2.9 |
| 291 | 13 | Female | NA | 3.1 |
| 292 | 2.3 | Female | NA | 3.3 |
| 293 | 1 | Female | NA | 1.6 |
| 294 | NA | Male | NA | 3.4 |
| 295 | 60 | Male | NA | 3.2 |
| 296 | 0.9 | Female | NA | 4.7 |
| 297 | 55 | Male | NA | 2.2 |
| 298 | 30 | Female | NA | 9.9 |
| 299 | 43 | Male | NA | 2.4 |
| 300 | 10 | Male | NA | 1.4 |
| 301 | 82 | Male | NA | 1.3 |
| 302 | 2.3 | Female | NA | 4 |
| 303 | 18 | Male | NA | 2.3 |
| 304 | 2.7 | Male | NA | 2.2 |
| 305 | 2 | Female | NA | 6.3 |
| 306 | 6 | Male | 35044 | NA |
| 307 | 5 | Male | 37331 | NA |
| 308 | 1 | Female | 5349 | 1.3 |
| 309 | 26 | Male | 66160 | NA |
| 310 | 41 | Male | 43678 | NA |
| 311 | 77 | Male | 11113 | NA |
| 312 | NA | Male | 188925 | NA |
| 313 | 1.2 | Female | 43837 | 1 |
| 314 | 49 | Male | 223300 | NA |
| 315 | 27 | Male | 15058 | NA |
| 316 | 4 | Female | 19515 | NA |
| 317 | 24 | Male | 16361 | NA |
| 318 | 1 | Female | 15376 | NA |
| 319 | 54 | Female | 9046 | NA |
| 320 | 66 | Female | 82495 | NA |
| 321 | 46 | Male | 26168 | NA |
| 322 | NA | Female | 19202 | NA |
| 323 | 69 | Male | 135945 | NA |
| 324 | 41 | Male | 64130 | 2.8 |
| 325 | 2 | Male | 23307 | 1.6 |
| 326 | 53 | Female | 4074 | NA |
| 327 | 58 | Male | 97120 | NA |
| 328 | 11 | Male | 5833 | NA |
| 329 | 34 | Female | 5186 | NA |
| 330 | 36 | Male | 24712 | NA |
| 331 | 43 | Male | 2497 | NA |
| 332 | 1.9 | Male | 167590 | NA |
| 333 | NA | Male | 42996 | NA |
| 334 | 43 | Male | 3652 | NA |
| 335 | 1 | Male | 37389 | 1.6 |
| 336 | 65 | Male | 7935 | NA |
| 337 | 22 | Male | 3657 | NA |
| 338 | 4 | Male | 298140 | 3.5 |
| 339 | 30 | Female | 1145 | NA |
| 340 | 30 | Female | 4156 | 3.8 |
| 341 | 1 | Female | 5888 | NA |
| 342 | 15 | Male | 24975 | 5.2 |
| 343 | 34 | Male | 20672 | NA |
| 344 | 0.9 | Male | 46472 | 1.9 |
| 345 | 37 | Female | 46010 | NA |
| 346 | 32 | Male | 39348 | NA |
| 347 | 52 | Female | 20680 | NA |
| 348 | 53 | Male | 21340 | NA |
| 349 | 18 | Female | 2963 | NA |
| 350 | 1 | Male | 29970 | 1.63 |
| 351 | 41 | Male | 4751 | NA |
| 352 | 68 | Male | 194895 | NA |
| 353 | 3 | Female | 73795 | 5.5 |
| 354 | 6.6 | Female | 12344 | NA |
| 355 | 62 | Female | 15798 | NA |
| 356 | 79 | Female | 10184 | NA |
| 357 | 56 | Female | 111595 | NA |
| 358 | 3 | Female | 27709 | NA |
| 359 | 67 | Female | 33688 | NA |
| 360 | 17 | Male | 1853 | NA |
| 361 | 42 | Female | 24067 | NA |
| 362 | 69 | Male | 19980 | NA |
| 363 | 3 | Female | 207335 | NA |
| 364 | 1.7 | Male | 77820 | 2 |
| 365 | 4 | Male | 98210 | 3 |
| 366 | 16 | Female | 28678 | NA |
| 367 | NA | Male | 127590 | NA |
| 368 | 3.7 | Female | 15463 | 2.5 |
| 369 | 6 | Male | 9713 | NA |
| 370 | 65 | Female | 35583 | NA |
| 371 | 3 | Male | 4033 | 4.7 |
| 372 | 5 | Male | 50450 | 2.8 |
| 373 | NA | Male | 37295 | NA |
| 374 | 1 | Male | 19794 | 6.9 |
| 375 | 51 | Male | 142495 | NA |
| 376 | 3 | Male | 42727 | 1.8 |
| 377 | 38 | Male | 25000 | NA |
| 378 | 4 | Female | 33263 | 2.2 |
| 379 | 45 | Male | 11725 | NA |
| 380 | 2 | Female | 122190 | NA |
| 381 | 24 | Female | 16740 | NA |
| 382 | 57 | Female | 35799 | NA |
| 383 | 4 | Male | 198615 | 2.9 |
| 384 | 62 | Female | 13635 | 3.9 |
| 385 | 59 | Female | 166505 | NA |
| 386 | 30 | Male | 21632 | NA |
| 387 | 23 | Female | 54625 | 3.6 |
| 388 | 3 | Male | 1736 | NA |
| 389 | 5.5 | Male | 228755 | 2.3 |
| 390 | 2.6 | Female | 3142 | NA |
| 391 | 6 | Male | 31092 | 2.6 |
| 392 | 3 | Male | 109865 | NA |
| 393 | 2 | Male | 20476 | 3.9 |
| 394 | 1.5 | Male | 6565 | NA |
| 395 | 45 | Male | 110000 | NA |
| 396 | 49 | Male | 28039 | NA |
| 397 | 34 | Female | 20475 | NA |
| 398 | 25 | Male | 2786 | NA |
| 399 | 15 | Female | 33384 | NA |
| 400 | 45 | Male | 30231 | NA |
| 401 | 60 | Female | 42771 | NA |
| 402 | 1 | Male | 9884 | NA |
| 403 | 6 | Female | 25111 | 3.4 |
| 404 | 0.9 | Female | 30476 | NA |
| 405 | 43 | Male | 53993 | NA |
| 406 | 17 | Male | 31320 | 2.3 |
| 407 | 0.1 | Female | 7817 | NA |
| 408 | 78 | Female | 7038 | NA |
| 409 | 38 | Male | 27820 | NA |
| 410 | 36 | Male | 30527 | NA |
| 411 | 61 | Male | 11097 | NA |
| 412 | 56 | Female | 31715 | NA |
| 413 | 45 | Female | 6331 | NA |
| 414 | 57 | Female | 139085 | NA |
| 415 | 28 | Male | 101 | NA |
| 416 | NA | Male | 6442 | NA |
| 417 | 14 | Male | 7831 | NA |
| 418 | 66 | Male | 22666 | NA |
| 419 | NA | Male | 10989 | NA |
| 420 | 30 | Female | 70700 | NA |
| 421 | 37 | Female | 14538 | NA |
| 422 | 2.7 | Female | 560 | NA |
| 423 | 23 | Male | 1980 | NA |
| 424 | 30 | Male | 21443 | NA |
| 425 | 55 | Female | 36465 | NA |
| 426 | 1.8 | Female | 12188 | NA |
| 427 | 1.2 | Female | 28631 | 2.8 |
| 428 | 28 | Female | 14616 | NA |
| 429 | 35 | Male | 48330 | NA |
| 430 | 37 | Male | 294375 | NA |
| 431 | 5 | Female | 42620 | NA |
| 432 | 4.3 | Male | 219830 | NA |
| 433 | 1 | Female | 23640 | NA |
| 434 | 20 | Female | 144615 | NA |
| 435 | NA | Male | 13603 | NA |
| 436 | 70 | Male | 3756 | NA |
| 437 | 48 | Male | 14448 | NA |
| 438 | 79 | Male | 135970 | NA |
| 439 | 58 | Female | 15095 | NA |
| 440 | 45 | Male | 37529 | NA |
| 441 | 63 | Female | 7825 | NA |
| 442 | NA | Male | 3986 | NA |
| 443 | 30 | Male | 23647 | NA |
| 444 | 55 | Male | 90235 | NA |
| 445 | 32 | Female | 105570 | NA |
| 446 | 1 | Female | 15894 | NA |
| 447 | 2.9 | Male | 2390 | NA |
| 448 | 7.7 | Male | 217550 | 3.7 |
| 449 | 0.1 | Male | 38787 | NA |
| 450 | 1.7 | Male | 9119 | 1.1 |
| 451 | 28 | Male | 3513 | NA |
| 452 | 15 | Female | 16830 | NA |
| 453 | 21 | Female | 143745 | NA |
| 454 | 16 | Male | 21049 | NA |
| 455 | 3.5 | Female | 86695 | NA |
| 456 | 45 | Male | 1476 | NA |
| 457 | 56 | Female | 17146 | NA |
| 458 | 2 | Male | 29131 | 2.8 |
| 459 | 19 | Male | 110430 | NA |
| 460 | 69 | Male | 112115 | NA |
| 461 | 50 | Female | 17989 | NA |
| 462 | 10.3 | Female | 6443 | NA |
| 463 | 1 | Female | 4413 | 1.4 |
| 464 | 12 | Female | 34405 | 2.4 |
| 465 | 38 | Male | 18525 | NA |
| 466 | 33 | Female | 5066 | NA |
| 467 | 1.3 | Female | 28168 | NA |
| 468 | 1.6 | Male | 13942 | NA |
| 469 | 31 | Female | 18703 | NA |
| 470 | 54 | Male | 25582 | 1.5 |
| 471 | 32 | Female | 45570 | NA |
| 472 | 10 | Male | 303470 | NA |
| 473 | 3.2 | Male | 7822 | 3.5 |
| 474 | 34 | Male | 36291 | NA |
| 475 | NA | Male | 10305 | NA |
| 476 | 1.2 | Female | 31710 | NA |
| 477 | 2.6 | Female | 101500 | 1 |
| 478 | 40 | Female | 16637 | NA |
| 479 | 11 | Male | 10351 | NA |
| 480 | 13 | Female | 5727 | 2.7 |
| 481 | 55 | Female | 2384 | NA |
| 482 | 12 | Male | 154875 | NA |
| 483 | 27 | Female | 2124 | NA |
| 484 | 40 | Male | 14068 | NA |
| 485 | 76 | Male | 40492 | NA |
| 486 | 27 | Female | 15143 | NA |
| 487 | 94 | Female | 23550 | NA |
| 488 | 69 | Female | 17871 | NA |
| 489 | 37 | Female | 27762 | NA |
| 490 | 0.9 | Female | 27405 | 2 |
| 491 | 32 | Female | 4515 | NA |
| 492 | 73 | Male | 132755 | NA |
| 493 | NA | Male | 116970 | NA |
| 494 | 51 | Female | 152155 | NA |
| 495 | 34 | Female | 11054 | NA |
| 496 | 61 | Male | 215520 | NA |
| 497 | 15 | Male | 107670 | NA |
| 498 | 20 | Male | 97740 | NA |
| 499 | 1.5 | Male | 43890 | NA |
| 500 | 72 | Female | 264310 | NA |
| 501 | 3 | Female | 12956 | 2.8 |
| 502 | 3.5 | Female | 20111 | NA |
| 503 | 0.6 | Male | 9470 | NA |
| 504 | 2.2 | Male | 1564 | NA |
| 505 | 29 | Female | 28669 | NA |
| 506 | 16 | Female | 5050 | 2.8 |
| 507 | 35 | Male | 10151 | NA |
| 508 | 1 | Female | 161330 | 2.9 |
| 509 | 59 | Male | 7372 | 2 |
| 510 | 37 | Female | 12056 | NA |
| 511 | 53 | Female | 31634 | NA |
| 512 | NA | Male | 2347 | 5 |
| 513 | 29 | Male | 7911 | NA |
| 514 | 40 | Male | 127645 | NA |
| 515 | 50 | Male | 42700 | NA |
| 516 | 75 | Male | 17454 | NA |
| 517 | 1.2 | Male | 10189 | 2.8 |
| 518 | 3 | Male | 3858 | 2.3 |
| 519 | 8 | Female | 8761 | NA |
| 520 | 4 | Female | 6224 | NA |
| 521 | 27 | Male | 26813 | NA |
| 522 | 0.1 | Female | 7211 | NA |
| 523 | 3.4 | Male | 1253 | NA |
| 524 | 2 | Female | 86705 | 6.3 |
| 525 | 4 | Female | 18856 | NA |
| 526 | 32 | Male | 4552 | NA |
| 527 | 30 | Male | 69692 | NA |
| 528 | 7 | Male | 23845 | 1.7 |
| 529 | 29 | Female | 11776 | NA |
| 530 | 47 | Male | 82020 | NA |
| 531 | 31 | Male | 42101 | NA |
| 532 | 22 | Male | 80205 | NA |
| 533 | 38 | Male | 19389 | NA |
| 534 | 17 | Female | 1466 | NA |
| 535 | 46 | Female | 5295 | NA |
| 536 | 6 | Female | 9322 | 3.2 |
| 537 | 23 | Female | 16726 | NA |
| 538 | 56 | Female | 2272 | NA |
| 539 | 86 | Female | 101405 | NA |
| 540 | 35 | Female | 6506 | NA |
| 541 | 34 | Female | 10428 | NA |
| 542 | 24 | Male | 196365 | NA |
| 543 | 29 | Male | 5004 | NA |
| 544 | 47 | Male | 51222 | 2.8 |
| 545 | 0.7 | Female | 43762 | NA |
| 546 | 48 | Male | 4475 | NA |
| 547 | 35 | Male | 10765 | NA |
| 548 | 6 | Female | 3906 | 3.2 |
| 549 | 14 | Female | 22939 | NA |
| 550 | 6.1 | Female | 22685 | 2.7 |
| 551 | 60 | Female | 141445 | NA |
| 552 | 34 | Female | 29139 | NA |
| 553 | 57 | Male | 12762 | NA |
| 554 | 3 | Male | 108360 | NA |
| 555 | 29 | Female | 45958 | NA |
| 556 | 1.7 | Male | 93060 | 3.9 |
| 557 | 73 | Male | 89123 | NA |
| 558 | 78 | Male | 33835 | NA |
| 559 | 32 | Female | 5711 | NA |
| 560 | 0.6 | Male | 16139 | 2.3 |
| 561 | 23 | Male | 238890 | 2.9 |
| 562 | 0.9 | Male | 19652 | 2.5 |
| 563 | 21 | Male | 9652 | NA |
| 564 | 19 | Male | 121825 | NA |
| 565 | 3 | Female | 86030 | 2.2 |
| 566 | 39 | Male | 14217 | NA |
| 567 | 17 | Male | 7776 | NA |
| 568 | 60 | Male | 63740 | NA |
| 569 | 21 | Female | 16807 | NA |
| 570 | 60 | Female | 43921 | NA |
| 571 | 43 | Female | 38440 | NA |
| 572 | 8 | Male | 15331 | 2.7 |
| 573 | 10 | Male | 18541 | 2.9 |
| 574 | 4 | Female | 117885 | 3 |
| 575 | 1 | Male | 165925 | NA |
| 576 | 66 | Male | 87260 | NA |
| 577 | 1 | Male | 15533 | 2.5 |
| 578 | 44 | Female | 13777 | NA |
| 579 | 47 | Male | 33145 | NA |
| 580 | 51 | Female | 13913 | NA |
| 581 | 56 | Male | 47684 | NA |
| 582 | 40 | Male | 10351 | NA |
| 583 | 64 | Male | 85400 | 4.6 |
| 584 | 4.5 | Female | 259715 | NA |
| 585 | 15 | Female | 34150 | NA |
| 586 | 3 | Female | 23462 | NA |
| 587 | 65 | Female | 12137 | NA |
| 588 | 32 | Female | 14951 | NA |
| 589 | 14.9 | Male | 80180 | NA |
| 590 | 9.5 | Male | 13191 | NA |
| 591 | 84 | Female | 122570 | NA |
| 592 | 3.9 | Male | 40782 | NA |
| 593 | 33 | Female | 11921 | NA |
| 594 | NA | Male | 8874 | NA |
| 595 | 37 | Female | 7219 | NA |
| 596 | 44 | Male | 212530 | NA |
| 597 | 50 | Male | 178260 | NA |
| 598 | 15 | Male | 42052 | 2.9 |
| 599 | 72 | Male | 43416 | NA |
| 600 | 9 | Male | 10096 | NA |
| 601 | 25 | Female | 10798 | NA |
| 602 | 37 | Female | 11849 | NA |
| 603 | NA | Female | 11743 | NA |
| 604 | 1 | Male | 6239 | 1.5 |
| 605 | 53 | Male | 20292 | NA |
| 606 | 52 | Female | 6822 | NA |
| 607 | 24 | Male | 30250 | NA |
| 608 | 57 | Male | 43594 | NA |
| 609 | 63 | Male | 1976 | NA |
| 610 | 32 | Female | 178590 | NA |
| 611 | 69 | Female | 26193 | NA |
| 612 | 6 | Male | 2286 | NA |
| 613 | 26 | Female | 40085 | NA |
| 614 | 12 | Male | 6449 | 6.4 |
| 615 | 27 | Female | 7833 | NA |
| 616 | 58 | Male | 37937 | NA |
| 617 | 31 | Male | 29994 | NA |
| 618 | 57 | Male | 10648 | NA |
| 619 | 66 | Female | 7678 | NA |
| 620 | 29 | Female | 4544 | NA |
| 621 | NA | Male | 124880 | NA |
| 622 | 35 | Male | 38642 | NA |
| 623 | 70 | Male | 163175 | NA |
| 624 | 33 | Male | 280215 | NA |
| 625 | 4 | Male | 35038 | 1.8 |
| 626 | 21 | Female | 20903 | NA |
| 627 | 63 | Female | 15314 | NA |
| 628 | 63 | Female | 34920 | NA |
| 629 | 35 | Male | 19848 | NA |
| 630 | 4 | Female | 212845 | 4.5 |
| 631 | 3.8 | Male | 18492 | 7.8 |
| 632 | 12 | Male | 158565 | 1.9 |
| 633 | 3 | Male | 35113 | 2.8 |
| 634 | 36 | Male | 7658 | 3.3 |
| 635 | 55 | Female | 12732 | NA |
| 636 | 51 | Female | 135090 | NA |
| 637 | 14 | Male | 22301 | 2.4 |
| 638 | 59 | Female | 2212 | NA |
| 639 | 59 | Male | 134410 | NA |
| 640 | 8 | Female | 20800 | 3.5 |
| 641 | 50 | Male | 15866 | NA |
| 642 | 20 | Female | 1540 | NA |
| 643 | 37 | Female | 26761 | NA |
| 644 | 1 | Female | 10143 | 1.4 |
| 645 | 10 | Female | 179315 | 2 |
| 646 | 67 | Male | 30250 | NA |
| 647 | NA | Male | 42058 | NA |
| 648 | 44 | Male | 14320 | NA |
| 649 | 10 | Female | 9312 | 2.4 |
| 650 | 34 | Male | 38241 | NA |
| 651 | 53 | Male | 55835 | NA |
| 652 | 45 | Female | 2979 | NA |
| 653 | 26 | Female | 6827 | NA |
| 654 | 3 | Male | 85325 | 5.7 |
| 655 | NA | Male | 931 | NA |
| 656 | 67 | Female | 1203 | NA |
| 657 | 52 | Male | 200315 | NA |
| 658 | 33 | Female | 31809 | NA |
| 659 | 1 | Female | 9055 | 4.4 |
| 660 | 49 | Female | 95960 | NA |
| 661 | 32 | Male | 11432 | NA |
| 662 | 53 | Male | 13071 | NA |
| 663 | 76 | Male | 40225 | NA |
| 664 | 1 | Female | 25666 | 1.9 |
| 665 | 72 | Male | 9574 | NA |
| 666 | 68 | Male | 17839 | NA |
| 667 | 36 | Male | 40516 | NA |
| 668 | 21 | Male | 25803 | NA |
| 669 | 62 | Male | 18200 | NA |
| 670 | 62 | Female | 2477 | NA |
| 671 | 70 | Male | 37199 | NA |
| 672 | 20 | Female | 7637 | NA |
| 673 | 69 | Male | 8939 | NA |
| 674 | 24 | Male | 60565 | NA |
| 675 | 3 | Female | 13802 | 3.7 |
| 676 | 58 | Male | 225650 | NA |
| 677 | 69 | Male | 124510 | NA |
| 678 | 43 | Female | 31884 | NA |
| 679 | 3 | Male | 73885 | 1.8 |
| 680 | 2 | Female | 43451 | 2.6 |
| 681 | 69 | Male | 8748 | NA |
| 682 | 63 | Male | 19183 | NA |
| 683 | 46 | Male | 39646 | NA |
| 684 | 65 | Male | 42057 | NA |
| 685 | 66 | Female | 4858 | NA |
| 686 | 34 | Male | 15315 | NA |
| 687 | 58 | Male | 34344 | NA |
| 688 | 35 | Male | 37806 | NA |
| 689 | 11 | Female | 12882 | 3.2 |
| 690 | 40 | Male | 151025 | NA |
| 691 | 43 | Male | 249595 | NA |
| 692 | 63 | Female | 2245 | NA |
| 693 | 75 | Male | 12908 | NA |
| 694 | 2.2 | Male | 23606 | 4.6 |
| 695 | 24 | Male | 23335 | NA |
| 696 | 28 | Female | 119550 | NA |
| 697 | 24 | Male | 3643 | NA |
| 698 | 41 | Male | 139890 | NA |
| 699 | 2.2 | Male | 3971 | 2 |
| 700 | 30 | Male | 6078 | NA |
| 701 | 32 | Female | 13989 | NA |
| 702 | 63 | Female | 13063 | NA |
| 703 | NA | Female | 13154 | NA |
| 704 | 30 | Male | 17338 | NA |
| 705 | 23 | Male | 206455 | 3.7 |
| 706 | 32 | Male | 27777 | NA |
| 707 | 22 | Male | 1486 | NA |
| 708 | 71 | Male | 1793 | NA |
| 709 | 37 | Male | 33519 | 4.6 |
| 710 | 0.5 | Female | 16186 | 3 |
| 711 | NA | Male | 5310 | NA |
| 712 | 57 | Female | 29307 | NA |
| 713 | 26 | Male | 36575 | NA |
| 714 | 26 | Female | 18339 | NA |
| 715 | 27 | Female | 8114 | NA |
| 716 | 15 | Female | 66390 | 2.5 |
| 717 | 49 | Female | 5745 | 3.8 |
| 718 | 44 | Female | 41617 | NA |
| 719 | 24 | Male | 24167 | NA |
| 720 | 49 | Female | 6038 | NA |
| 721 | 59 | Male | 4436 | NA |
| 722 | 48 | Male | 47570 | NA |
| 723 | NA | Male | 37833 | NA |
| 724 | NA | Female | 8002 | NA |
| 725 | 34 | Male | 25518 | NA |
| 726 | 52 | Male | 22951 | NA |
| 727 | 70 | Female | 9133 | 5 |
| 728 | 32 | Female | 24055 | NA |
| 729 | 24 | Male | 546300 | NA |
| 730 | 57 | Male | 42094 | NA |
| 731 | 72 | Female | 6201 | NA |
| 732 | 0.2 | Male | 25445 | 1.3 |
| 733 | 46 | Male | 19355 | NA |
| 734 | 13 | Male | 60565 | 1.7 |
| 735 | NA | Male | 4218 | NA |
| 736 | 39 | Female | 19150 | NA |
| 737 | 37 | Female | 16246 | NA |
| 738 | 35 | Female | 23226 | NA |
| 739 | 45 | Female | 34814 | NA |
| 740 | 21 | Female | 28183 | NA |
| 741 | 67 | Male | 52866 | NA |
| 742 | 72 | Male | 59805 | NA |
| 743 | 24 | Male | 29617 | NA |
| 744 | 50 | Female | 10221 | NA |
| 745 | 27 | Male | 126560 | NA |
| 746 | 65 | Male | 40351 | NA |
| 747 | 63 | Female | 68455 | NA |
| 748 | 45 | Female | 17845 | NA |
| 749 | 1 | Female | 186110 | 3 |
| 750 | 60 | Male | 14712 | NA |
| 751 | 32 | Female | 3637 | NA |
| 752 | 24 | Female | 11133 | NA |
| 753 | 39 | Female | 8954 | NA |
| 754 | 1.7 | Male | 72790 | 1.2 |
| 755 | 32 | Female | 3781 | NA |
| 756 | 53 | Male | 40100 | NA |
| 757 | 27 | Male | 26498 | NA |
| 758 | 48 | Male | 26036 | NA |
| 759 | 59 | Female | 9554 | NA |
| 760 | 27 | Male | 116115 | NA |
| 761 | 56 | Male | 21786 | NA |
| 762 | 67 | Male | 40926 | NA |
| 763 | 83 | Male | 1076 | NA |
| 764 | 67 | Female | 1022 | NA |
| 765 | 37 | Male | 42092 | NA |
| 766 | 47 | Male | 90905 | 4.6 |
| 767 | 0.2 | Male | 20581 | 3.7 |
| 768 | 55 | Male | 41869 | NA |
| 769 | 71 | Female | 6191 | NA |
| 770 | 33 | Male | 28926 | NA |
| 771 | 65 | Male | 37665 | NA |
| 772 | NA | Female | 110754 | NA |
| 773 | 67 | Female | 18631 | NA |
| 774 | 65 | Female | 3838 | NA |
| 775 | 41 | Male | 18238 | NA |
| 776 | 15 | Female | 28512 | 3.7 |
| 777 | NA | Female | 40410 | NA |
| 778 | 44 | Male | 38220 | NA |
| 779 | NA | Male | 41463 | NA |
| 780 | 33 | Female | 6749 | NA |
| 781 | 23 | Male | 7676 | NA |
| 782 | 22 | Male | 8281 | NA |
| 783 | 33 | Female | 5999 | NA |
| 784 | 61 | Male | 42995 | NA |
| 785 | 55 | Male | 7229 | NA |
| 786 | 21 | Male | 17776 | NA |
| 787 | 60 | Female | 137735 | NA |
| 788 | 22 | Male | 28380 | NA |
| 789 | 76 | Male | 38760 | NA |
| 790 | 28 | Female | 10724 | NA |
| 791 | 32 | Male | 7179 | NA |
| 792 | 26 | Female | 34769 | NA |
| 793 | 83 | Male | 5669 | NA |
| 794 | 4 | Male | 7722 | 1.8 |
| 795 | 65 | Male | 3905 | NA |
| 796 | 19 | Female | 42268 | 2.4 |
| 797 | 81 | Female | 42973 | NA |
| 798 | 65 | Female | 53200 | NA |
| 799 | 9 | Male | 4040 | 2 |
| 800 | 54 | Female | 12186 | 3 |
| 801 | 37 | Male | 9662 | NA |
| 802 | 20 | Male | 4605 | NA |
| 803 | 75 | Female | 106720 | NA |
| 804 | 23 | Male | 39750 | NA |
| 805 | 4 | Female | 32503 | 1.8 |
| 806 | 55 | Female | 26617 | NA |
| 807 | 6 | Male | NA | 3.5 |
| 808 | 8 | Female | NA | 2.4 |
| 809 | NA | Male | NA | 3 |
| 810 | 15 | Female | NA | 4.2 |
| 811 | 8 | Female | 34472 | 5.7 |
| 812 | NA | Male | 37719 | 3.4 |
| 813 | 8 | Female | NA | 4.3 |
| 814 | 53 | Female | 36547 | NA |
| 815 | 5 | Female | 28977 | 3.1 |
| 816 | 4.2 | Female | NA | 4.8 |
| 817 | 11 | Male | NA | 2.2 |
| 818 | 5 | Female | NA | 5 |
| 819 | 74 | Male | 28016 | 2.5 |
| 820 | 29 | Male | NA | 2.4 |
| 821 | 24 | Female | 17859 | 2.1 |
| 822 | 2 | Male | NA | 1.9 |
| 823 | 1.9 | Male | 11091 | 2.7 |
| 824 | 42 | Female | 13437 | 2.5 |
| 825 | 2.9 | Male | NA | 4.4 |
| 826 | 59 | Male | 26809 | NA |
| 827 | 2.4 | Female | 40514 | 3.2 |
| 828 | 16 | Female | 29066 | 3.7 |
| 829 | 50 | Male | 63585 | 1.6 |
| 830 | NA | Male | 122515 | 2 |
| 831 | 3 | Female | NA | 4.4 |
| 832 | 61 | Male | 39519 | 2.8 |
| 833 | 55 | Male | 38000 | 7.2 |
| 834 | 4 | Male | 30964 | 2.5 |
| 835 | NA | Female | 33025 | 1.5 |
| 836 | 37 | Female | 58240 | 1.8 |
| 837 | 0.8 | Male | NA | 2.1 |
| 838 | 43 | Female | 19023 | NA |
| 839 | 56 | Male | NA | NA |
| 840 | 27 | Female | NA | 2.8 |
| 841 | 11 | Male | NA | 1.6 |
| 842 | NA | Male | 36879 | NA |
| 843 | 58 | Male | NA | 1.5 |
| 844 | 49 | Male | 10287 | 2.4 |
| 845 | 4 | Male | 111835 | 3.8 |
| 846 | 7 | Male | 283050 | 4 |
| 847 | 57 | Male | 42238 | 3.2 |
| 848 | NA | Male | 41017 | 6 |
| 849 | 24 | Female | 31510 | 1.9 |
| 850 | 74 | Male | 24732 | 2.8 |
| 851 | 40 | Male | 54265 | 2.4 |
| 852 | 2.6 | Female | NA | 3.6 |
| 853 | NA | Male | 13062 | 3.1 |
| 854 | 28 | Female | 18538 | 2.8 |
| 855 | 39 | Male | NA | 3 |
| 856 | 1 | Female | NA | 1.8 |
| 857 | NA | Male | 2781 | 2.9 |
| 858 | 65 | Female | 8721 | 2.1 |
| 859 | 1.1 | Male | 36343 | 1.4 |
| 860 | 74 | Male | 42269 | 2.3 |
| 861 | 70 | Female | 204465 | 2.8 |
| 862 | 26 | Male | 154845 | 1.3 |
| 863 | 4.7 | Female | NA | 3.5 |
| 864 | 2 | Female | 25692 | 3.2 |
| 865 | NA | Female | 40275 | 1.8 |
| 866 | 57 | Female | 14712 | 3.8 |
| 867 | NA | Male | NA | 1.6 |
| 868 | NA | Male | 304810 | 1.7 |
| 869 | NA | Male | 31251 | 1.6 |
| 870 | 41 | Male | 21911 | NA |
| 871 | 1 | Male | NA | 1.9 |
| 872 | NA | Female | NA | 3.4 |
| 873 | 2 | Male | NA | 2.1 |
| 874 | 5 | Male | 16375 | 1.5 |
| 875 | 20 | Male | 3324 | 3.6 |
| 876 | 24 | Male | 9637 | NA |
| 877 | NA | Male | 13304 | 3.2 |
| 878 | NA | Female | 10078 | 4 |
| 879 | NA | Male | 32292 | 2.3 |
| 880 | 67 | Female | 98260 | 4.2 |
| 881 | 44 | Female | 27519 | 1.3 |
| 882 | 39 | Male | 20917 | 1.8 |
| 883 | 2 | Male | NA | 1.8 |
| 884 | 7 | Female | NA | 4.5 |
| 885 | 57 | Female | 41115 | 1.3 |
| 886 | 16 | Female | NA | 2.2 |
| 887 | 52 | Female | 35082 | 3.1 |
| 888 | 47 | Female | 23015 | 2.3 |
| 889 | 8 | Female | NA | 3.2 |
| 890 | 0.2 | Female | NA | 3.4 |
| 891 | 13 | Male | NA | 1.3 |
| 892 | 11 | Female | NA | 2 |
| 893 | NA | Male | NA | 2.8 |
| 894 | 55 | Male | 8436 | 1.7 |
| 895 | 33 | Female | NA | 1.5 |
| 896 | 1.7 | Female | NA | 3.8 |
| 897 | 49 | Male | NA | 1.5 |
| 898 | 17 | Male | 97260 | 4.9 |
| 899 | 59 | Male | 180985 | 2.3 |
| 900 | 33 | Female | 24969 | NA |
| 901 | 60 | Male | NA | 2.3 |
| 902 | NA | Male | 43403 | 2.2 |
| 903 | 46 | Female | 37562 | 1.8 |
| 904 | 47 | Male | 39742 | 3.8 |
| 905 | 33 | Female | 11968 | 2.6 |
| 906 | 5 | Female | NA | NA |
| 907 | 26 | Male | 10379 | 6.2 |
| 908 | NA | Male | NA | 1.8 |
| 909 | 37 | Male | 39523 | 4 |
| 910 | 41 | Female | 6153 | 3.8 |

NA, not available.
